# Integrative Transcriptomic, Network, and Machine Learning Analyses Identify Genistein and Resveratrol-Associated Therapeutic Targets in Alzheimer’s Disease

**DOI:** 10.1007/s12035-026-05973-y

**Published:** 2026-06-02

**Authors:** Murat Isıyel, Hamid Ceylan, Yeliz Demir

**Affiliations:** 1 Faculty of Science, Department of Molecular Biology and Genetics, Atatürk University, Erzurum, Türkiye; 2 East Anatolian High Technology Research and Application Center (DAYTAM), Atatürk University, Erzurum, 25240 Türkiye; 3 Department of Pharmacy Services, Nihat Delibalta Göle Vocational High School, Ardahan University, Ardahan, 75700 Türkiye; 4 Faculty of Science, Department of Chemistry, Atatürk University, Erzurum, Türkiye

**Keywords:** Alzheimer’s disease, Machine learning, Transcriptomics, Genistein, Resveratrol

## Abstract

Alzheimer’s disease (AD) is a multifactorial neurodegenerative disorder characterized by complex molecular alterations across multiple brain regions. In this study, we applied an integrative systems-level framework combining multi-region transcriptomic analysis, protein–protein interaction (PPI) network topology, machine learning–based validation, and in silico approaches to identify robust and pharmacologically relevant molecular targets in AD. Gene expression data from four AD-related brain regions (entorhinal cortex, frontal cortex, hippocampus, and temporal cortex) were obtained from the GSE5281 dataset, yielding 467 genes consistently dysregulated across all regions. Network analysis identified a subset of downregulated hub genes predominantly associated with mitochondrial bioenergetics and proteostasis pathways. Intersection with experimentally curated targets of genistein and resveratrol prioritized four elite genes, COX5B, ENO1, HSP90AB1, and SDHB as shared and biologically central nodes. To assess the collective discriminative capacity of the identified gene set, a random forest classifier was trained using shared differentially expressed genes. The model demonstrated strong classification performance with a low out-of-bag error rate, while feature importance analysis identified SDHB as the most influential contributor, followed by COX5B and ENO1, indicating synergistic multigene effects rather than isolated biomarker behavior. Machine learning was applied as an integrative validation layer to reinforce biological relevance, rather than as a standalone diagnostic tool. Finally, molecular docking analyses revealed favorable binding affinities of genistein and resveratrol toward all four elite targets. Overall, these findings highlight a convergent mitochondrial–proteostasis dysfunction axis in AD and suggest COX5B, ENO1, HSP90AB1, and SDHB as promising multi-target nodes for polyphenol-based therapeutic strategies.

**Supplementary Information:**

The online version contains supplementary material available at 10.1007/s12035-026-05973-y.

## Introductıon

Alzheimer’s disease (AD) is the most prevalent neurodegenerative disorder and the leading cause of dementia worldwide, representing a major and escalating public health challenge due to population aging and the absence of curative therapies. Neuropathologically, AD is characterized by extracellular amyloid-β (Aβ) plaque deposition, intraneuronal neurofibrillary tangles composed of hyperphosphorylated tau, progressive synaptic dysfunction, and widespread neuronal loss, ultimately leading to irreversible cognitive decline and functional impairment [1, 2]. Though there has been significant advancement in biomarkers for AD and in defining stages of disease, current drug therapy remains primarily symptomatic and therefore there is an urgent need to find AD-relevant biological targets at the molecular level for disease modification and new therapeutic approaches [3].

AD pathology is the result of a complex interaction of multiple cellular stress pathways that converge on each other, including oxidative stress, impaired mitochondria function, dysfunctional protein homeostasis (proteostasis), neuroinflammatory responses, and aberrant synaptic signaling. The various processes will interact through a variety of brain regions including the entorhinal cortex, the hippocampus, and the frontal and temporal cortices, prior to the manifestation of clinically obvious symptoms [4, 5]. Therefore, an integrated analysis of gene expression in those parts of the brain most relevant to AD provides a potential method for identifying common and brain-region independent gene expression patterns which could be used as reliable diagnostic markers or as therapeutic targets in AD [6, 7].

In recent years, systems-level bioinformatics pipelines combining differential gene expression analysis, protein–protein interaction (PPI) network reconstruction, and topological hub gene prioritization have emerged as effective strategies to distill biologically meaningful targets from high-dimensional omics data. When coupled with curated compound-target databases and in silico validation frameworks, these approaches enable the rational identification of “elite” genes that are not only centrally positioned within disease-associated networks but are also pharmacologically accessible [8, 9]. Such integrative strategies have been successfully applied in oncology and are increasingly being adapted to neurodegenerative diseases to bridge the gap between molecular dysregulation and therapeutic feasibility.

Similar integrative transcriptomic and network-based approaches have been widely applied in AD to identify disease-associated gene signatures and key regulatory hubs [10]. For instance, studies have combined differential expression profiling with PPI network construction and hub gene ranking to uncover molecular mechanisms linked to synaptic dysfunction, mitochondrial impairment, and neuroinflammation in AD brain tissues [11]. Previous studies have applied integrated transcriptomic and network-based analyses to identify key regulatory genes and pathways involved in AD. While these approaches have successfully highlighted important molecular signatures, they have generally focused on single brain regions or specific datasets [12]. However, many of these studies have been limited to single brain regions or restricted datasets, which may overlook region-specific heterogeneity and shared cross-regional molecular signatures [13]. Therefore, more comprehensive multi-regional integrative analyses are still needed to better capture robust and reproducible gene networks underlying AD pathogenesis.

Phenolic phytochemicals have attracted considerable attention as potential multi-target agents in AD due to their pleiotropic biological activities, including antioxidant, anti-inflammatory, and neuroprotective effects. Unlike single-target drugs, these compounds can modulate multiple interconnected pathways implicated in AD pathogenesis, making them particularly attractive for complex, multifactorial disorders [14, 15]. Systematically intersecting AD-associated dysregulated genes with the known target repertoires of such compounds offers a rational framework for prioritizing candidate therapeutic targets that align with both disease biology and pharmacological action.

Although numerous phytochemicals and multi-target therapeutic candidates have recently been proposed for AD, genistein and resveratrol were specifically selected in the present study based on several methodological and biological considerations [16]. First, both compounds are among the most extensively investigated polyphenols in AD-related experimental research, with substantial evidence supporting their antioxidant, anti-inflammatory, mitochondrial-protective, and anti-amyloidogenic properties [16]. Second, unlike many phytochemicals with limited mechanistic characterization, genistein and resveratrol possess comparatively well-defined and experimentally curated target interaction profiles in public toxicogenomic resources such as CTD, enabling more reliable network-based target integration. Third, both compounds have been repeatedly implicated in multi-target modulation of interconnected AD-associated pathways, including oxidative stress, mitochondrial dysfunction, neuroinflammation, and proteostasis imbalance, making them particularly suitable candidates for systems-level and network pharmacology-oriented investigation. Therefore, the focus of the present study was not intended to represent the entire therapeutic landscape of AD phytochemicals, but rather to employ two biologically and pharmacologically well-characterized polyphenols as representative compounds for integrative target prioritization [17].

Recent advances in network pharmacology and network medicine have further emphasized the importance of evaluating therapeutic compounds based on their proximity to disease-associated molecular modules rather than single-target interactions alone. In AD, several therapeutic classes including polyphenols, flavonoids, kinase modulators, mitochondrial stabilizers, anti-inflammatory agents, and multi-target natural compounds have emerged as promising candidates within systems-level therapeutic frameworks [18]. Among these, genistein and resveratrol have consistently attracted attention because of their ability to simultaneously modulate oxidative stress, mitochondrial dysfunction, neuroinflammation, amyloid processing, and proteostasis-related pathways, all of which represent core interconnected components of AD-associated molecular networks. Although a formal network proximity analysis was beyond the scope of the present study, our integrative transcriptomic and PPI-based framework similarly aimed to prioritize compounds and targets positioned within biologically interconnected AD-relevant modules [19].

Genistein is an isoflavone from soy that has demonstrated extensive protective effects on AD as well as mechanisms such as powerful antioxidant and anti-inflammatory activity, modulation of estrogen receptor-mediated signaling, suppression of microglia activation, and protection of neurons against amyloid beta–induced neurotoxicity [20, 21]. Studies at the cellular and animal level have found that genistein can reduce amyloid burden, inhibit production of pro-inflammatory cytokines, and enhance cognitive function indicating genistein may be relevant to both the amyloid-driven and neuroinflammation portions of AD pathophysiology [22]. The specific molecular targets by which genistein will exert such effects on the human AD brain are also still largely undefined; thus, there is an unmet need for integrated target-driven analyses of genistein-responsive genes with respect to AD-associated changes in gene expression.

Resveratrol, a stilbene polyphenol present in the majority of grape varieties and berries, has similarly been identified as a major candidate for studies in the area of AD research as a result of its antioxidant, anti-amyloidogenic, and sirtuin-activating capabilities [23]. Preclinical studies have demonstrated that resveratrol can reduce Aβ aggregation, modulate tau phosphorylation, enhance mitochondrial function, and suppress neuroinflammatory signaling, partly through the activation of SIRT1-dependent pathways and the regulation of cellular stress responses [24]. Although clinical translation has been challenged by limited bioavailability and variable outcomes, resveratrol remains highly relevant as a molecular probe to interrogate AD-related pathways and to identify pharmacologically tractable targets within disease-associated networks (Turner et al. 25.

In this context, the present study applies an integrative bioinformatics framework to systematically identify AD-associated therapeutic biomarker candidates that intersect with the target landscapes of genistein and resveratrol. By leveraging multi-cohort transcriptomic datasets from AD-relevant brain regions, high-confidence PPI network analysis, hub gene prioritization, compound–gene intersection, and orthogonal in silico validation, including molecular docking, we aim to nominate a focused set of elite genes with potential relevance for AD pathogenesis and therapeutic modulation. This approach is designed to generate testable hypotheses and to provide a rational foundation for subsequent experimental validation of genistein and resveratrol-associated targets in AD.

## Material and Method

### Data Collection

Publicly accessible independent microarray datasets related to AD acquired from GEO (https://www.ncbi.nlm.nih.gov/geo/, accessed on December 17, 2025) [26]. In this study, the GSE5281 dataset was selected (filters were applied based on the study’s objectives, such as *Homo sapiens*, expression profiles, and Alzheimer) because it includes four major AD-associated brain regions: the entorhinal cortex, frontal cortex, hippocampus, and temporal cortex. Comprehensive features of the expression profile are presented in Table 1.

**Table 1:** Features of the datasets used in this study

	Sample size	Platform	Profile	References
GSE5281	Entorhinal cortex (10 AD sample, 13 control sample) Frontal cortex (18 AD sample, 11 control sample) Hippocampus (10 AD sample, 13 control sample) Temporal cortex (16 AD sample, 12 control sample)	GPL570 Affymetrix	mRNA	[27]

### Data Processing and Target Identification

DEGs between AD brain tissues and non-demented control brain tissues obtained from publicly available transcriptomic datasets were examined using the GEO2R tool (https://www.ncbi.nlm.nih.gov/geo2r, accessed on December 17, 2025). A *p*-value < 0.05 and log2FC ≥  + 1 were defined as thresholds for identifying upregulated genes. For downregulated genes, the cutoff value was set to log2FC ≤ −1. Given the high-dimensional nature of transcriptomic data, these thresholds were complemented by subsequent network-based and multi-step validation analyses to improve the robustness of candidate gene selection. Shared genes across all datasets were analyzed using a Venn diagram produced using the Multiple List Comparator web tool (http://molbiotools.com/listcompare.html, accessed on December 17, 2025). Target genes of the selected chemical substance (Genistein and Resveratrol) were determined using the Comparative Toxicogenomics Database (https://ctdbase.org/, accessed on December 17, 2025) [28, 29]. CTD integrates both experimentally validated and inferred interactions derived from multiple evidence sources; therefore, the retrieved gene sets were considered as a comprehensive pool of potential targets for downstream analysis. Shared genes across all substances were analyzed using a Venn diagram employing the Multiple List Comparator web tool. Volcano plots were created using the Prism software (GraphPad Software, San Diego, CA, USA). A *p*-value < 0.05 and |log2FC|> ± 1 were set as the cutoff values for determining significant DEGs.

### PPI Network Analysis

PPI network was constructed to evaluate the interactions among genes with significantly altered expression levels in AD brain tissues compared to non-demented control brain tissues and to identify hub genes [30, 31]. The PPI network was created using the Search Tool for Retrieval of Interacting Genes database (https://string-db.org/, accessed on December 17, 2025) [32]. A confidence score of ≥ 0.7 was applied to minimize false-positive interactions. The generated network was visualized and further analyzed using Cytoscape software version 3.10.3 [33, 34]. To determine hub genes, three topological analysis algorithms of the CytoHubba plugin (Maximum Neighborhood Component (MNC), Degree, and Edge Percolated Component (EPC)) of Cytoscape were used. Cluster analysis was used to identify densely connected gene modules and to support hub gene prioritization based on network topology.

### Gene Ontology and Pathway Enrichment Analysis

Enrichment analysis of overlapping DEGs was conducted using Gene Ontology (GO) and Kyoto Encyclopedia of Genes and Genomes (KEGG) pathway analyses [35]. Enrichment analyses were conducted using the ToppFun module of the ToppGene online bioinformatics resource (https://toppgene.cchmc.org/enrichment.jsp, accessed on December 17, 2025) [36]. Pathway enrichment results were filtered using an FDR-adjusted significance threshold, which is widely adopted in multiple testing correction frameworks to control for false discovery rate in high-dimensional gene set analyses.

### In Silico Comparison and Validations

#### Gene-Tissue Expression

The brain region–specific expression profiles of the elite genes (*COX5B*, *ENO1*, *HSP90AB1*, and *SDHB*) were further assessed using the AlzData database (http://www.alzdata.org/, accessed on December 17, 2025) [37, 38]. The Differential Expression module was queried with official HGNC gene symbols to compare transcript abundance between AD samples and non-demented controls across AD-relevant brain regions, including the entorhinal cortex, hippocampus, temporal cortex, and frontal cortex. For each gene and region, the resulting differential expression outputs (effect size estimates and associated statistical significance metrics, where provided by the platform) were retrieved from the interface, exported, and organized for subsequent synthesis with the GEO-derived findings.

#### Single-Cell Expression

To explore the cellular distribution of the same elite genes at single-cell resolution, the Single Cell Expression module of AlzData was used. Each gene symbol was individually queried, and cell-type stratified expression patterns were examined across major brain cell populations (e.g., neuronal and non-neuronal/glial lineages) as presented by the database. The resulting plots and tables were exported and used to contextualize the tissue-level differential expression signals by indicating whether observed changes could plausibly be attributable to particular cell types.

#### Protein Interaction Analysis

The HPA (Human Protein Atlas; https://www.proteinatlas.org/humanproteome/interaction, accessed on December 17, 2025) database was used to retrieve interaction data for the four elite genes (*COX5B*,* ENO1*,* HSP90AB1*, and* SDHB*). For each gene, the HPA “Interaction” module was queried with the protein-class visualization selected, and network images for human proteins were exported.

#### Molecular Docking

Molecular docking simulations were performed to investigate the binding interactions of genistein and resveratrol with selected mitochondrial- and metabolism-related protein targets using AutoDock Vina [39] with explicitly defined parameters to ensure reproducibility. The exhaustiveness parameter was set to 12 to provide sufficient conformational sampling of ligand orientations. For each ligand–protein pair, a maximum of nine binding poses were generated, and the conformation with the lowest binding free energy (kcal/mol) was selected as the most favorable binding mode. The three-dimensional crystal structures of COX5B, ENO1, HSP90AB1, and SDHB were retrieved from the Protein Data Bank (PDB). Prior to docking, all protein structures were prepared by removing co-crystallized ligands, ions, and water molecules, followed by the addition of polar hydrogen atoms and assignment of Gasteiger charges using AutoDock Tools (ADT).

The chemical structures of genistein and resveratrol were obtained from the PubChem database and energy-minimized before docking. Ligands were prepared by defining rotatable bonds and assigning appropriate partial charges. Docking grids were generated to encompass the active or binding regions of each protein, ensuring sufficient coverage of the ligand-accessible cavities. Grid box dimensions were selected to fully include the relevant binding pockets based on structural and functional considerations.

Docking calculations were conducted using AutoDock Vina with default parameters, and the exhaustiveness level was set to ensure reliable conformational sampling. For each ligand–protein pair, multiple binding poses were generated, and the conformation with the lowest binding free energy (kcal/mol) was selected as the most favorable binding mode. Docking scores were interpreted comparatively within the same experimental setup, and no external reference ligands were used for absolute binding affinity calibration. Post-docking analyses, including visualization of binding orientations and identification of key intermolecular interactions such as hydrogen bonds, π–π stacking, π–alkyl, π–cation, and electrostatic interactions, were carried out using molecular visualization tools. These analyses were used to elucidate the molecular basis of ligand–protein recognition and binding stability.

### Machine Learning–Based Classification Analysis

To evaluate the discriminative potential of the identified shared DEGs and to address concerns regarding potential overfitting, a supervised machine learning framework was implemented using the GSE5281 dataset. Only samples with unambiguous diagnostic annotation (AD or normal control) were retained. Probe-level expression values were mapped to official gene symbols using the GPL570 platform annotation file obtained from the GEO database. When multiple probes corresponded to the same gene, their expression values were aggregated using the mean to obtain a single gene-level measurement. A total of 467 shared DEGs were initially identified from the integrative transcriptomic analysis. Following probe-to-gene mapping and filtering based on gene availability in the expression matrix, all 467 genes were retained for downstream machine learning analysis. To improve model generalizability and mitigate overfitting, the dataset was randomly divided into training (80%) and testing (20%) subsets using a stratified sampling approach to preserve class distribution. A random forest classifier consisting of 1000 trees was trained on the training set using repeated stratified fivefold cross-validation. Model performance was evaluated on the independent test set using receiver operating characteristic (ROC) analysis, and the area under the curve (AUC) was used as the primary performance metric. To further assess model robustness and reduce dimensionality, additional models were constructed using biologically prioritized gene subsets. Specifically, a 13-gene panel derived from hub gene analysis based on the intersection of multiple CytoHubba algorithms applied to the protein–protein interaction network was evaluated. In addition, a minimal model based on four elite genes (COX5B, ENO1, HSP90AB1, and SDHB), selected based on network centrality and biological relevance, was also examined.

## Results

### Determination of DEGs

**Fig. 1:**
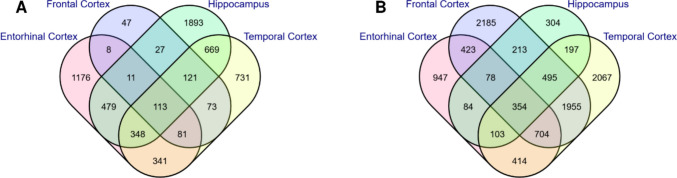
Venn diagrams showing common genes shared between all brain regions. **A** Upregulated genes. **B** Downregulated genes

**Fig. 2:**
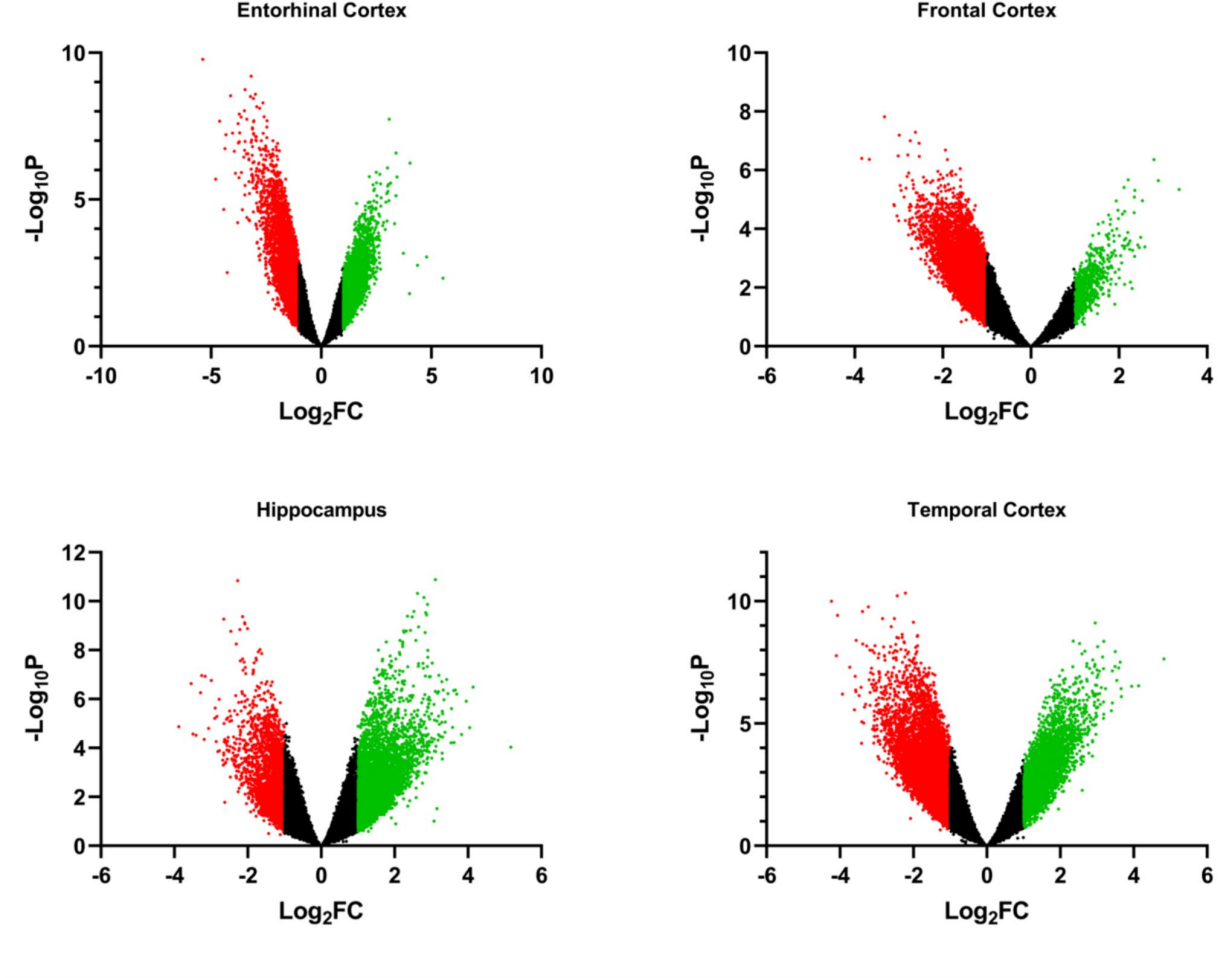
Volcano plots for the analyzed datasets revealed notable differences in expression between AD and control samples, based on a *p*-value < 0.05, and log2FC ≥  + 1 for upregulated and log2FC ≤ −1 for downregulated genes. Red dots represent downregulated genes and green dots represent upregulated genes, while black dots denote non-significant differences. FC, fold change

A total of 5664, 6888, 5189, and 8766 DEGs were identified in GSE5281 datasets for four brain regions, respectively (Table S1). Four hundred sixty-seven common DEGs were identified, comprising 113 upregulated and 354 downregulated genes shared across all brain regions (Fig. 1). The distribution of DEGs and overlapping genes identified as a result of the analysis of the dataset is summarized in Table S2. Volcano plots showing the distribution of DEGs created for each dataset are presented in Fig. 2.

### Chemical Gene Interaction Curation

**Fig. 3:**
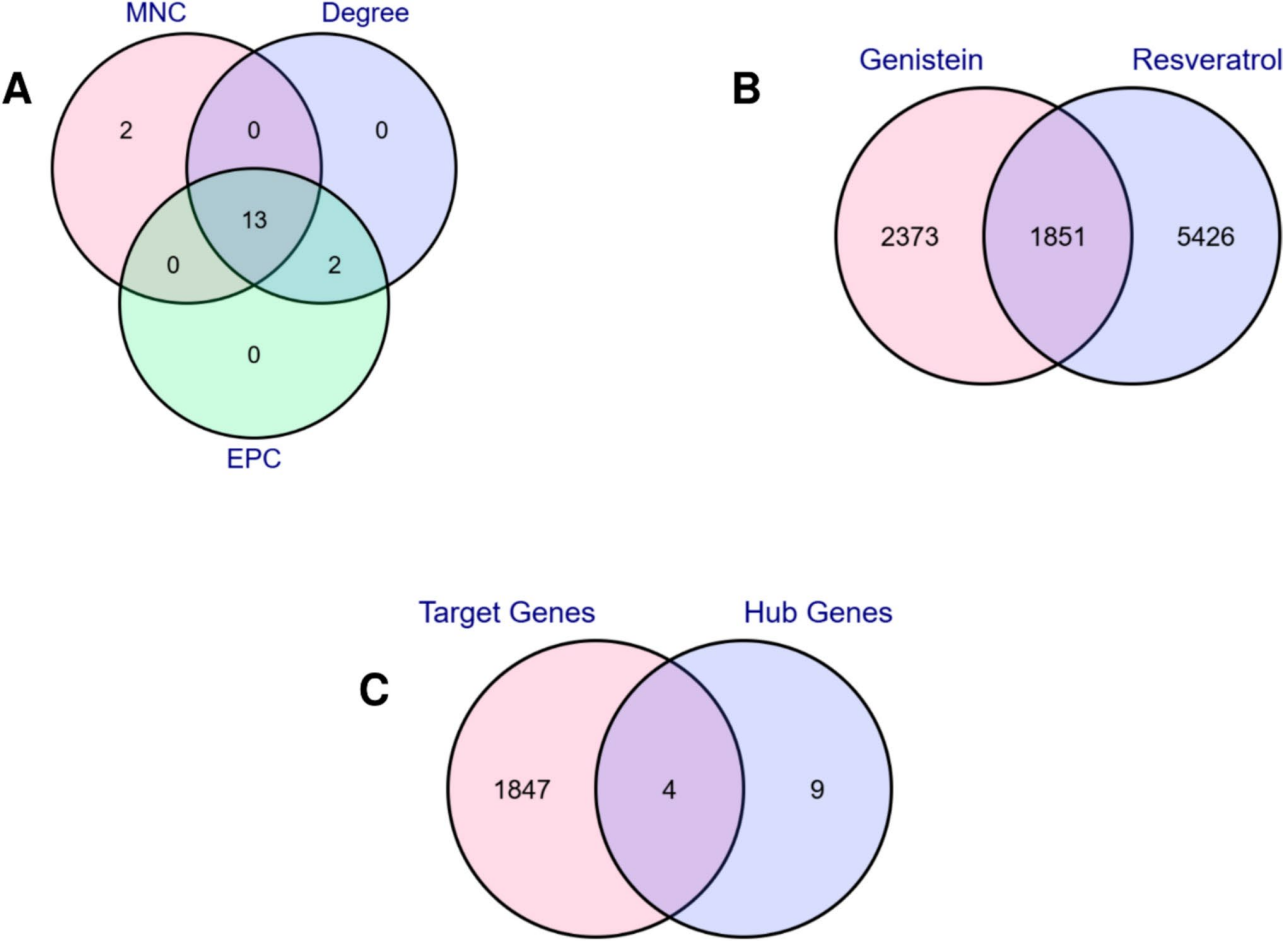
**A** Venn diagram analysis demonstrating the overlapping DEGs among the three algorithms of CytoHubba. **B** Venn diagram analysis demonstrating the overlapping target genes between two chemical compounds. **C** Venn diagram analysis demonstrating the overlapping four elite genes between target genes of two chemical compound and hub genes

To prioritize pharmacologically relevant targets in AD, the top 15 genes ranked by each of three topological algorithms MNC, Degree, and EPC in CytoHubba (Cytoscape) were compiled (Supplementary Table S3). Thirteen genes, consistently ranked across all methods, were identified as robust hub genes, including 13 downregulated genes (*ATP5A1*,* ATP5B*,* ATP5C1*,* COX5B*,* ENO1*,* HSP90AB1*,* NDUFA4*,* NDUFB5*,* NDUFS7*,* NDUFV1*,* SDHB*,* UQCRC1*,* UQCRC2*). These genes demonstrated strong topological centrality within the PPI network (Fig. 3A). To integrate potential therapeutic relevance, two bioactive agents, genistein and resveratrol, were selected based on their documented neuroprotective activity in AD models. Associated gene targets for each compound were retrieved from the Comparative Toxicogenomics Database (CTD). Intersection analysis revealed 1851 common target genes shared by two compounds (Supplementary Table S4; Fig. 3B). It should be noted that CTD-derived interactions include both direct and inferred associations, which may contribute to the large number of shared target genes observed. Therefore, the identified overlap should be interpreted as a reflection of potential shared biological pathways rather than strictly direct compound-gene interactions. A final cross-comparison between these compound-associated targets and the 13 hub genes identified four elite genes: *COX5B*,* ENO1*,* HSP90AB1*, and *SDHB* (Fig. 3C). These genes were prioritized as key AD-relevant candidates because they represent consistently downregulated, mitochondria, and proteostasis-associated hubs within the AD PPI network and also overlap with the experimentally curated target space of both genistein and resveratrol, supporting their potential pharmacological tractability in AD.

### PPI Network and Cluster Analysis

**Fig. 4:**
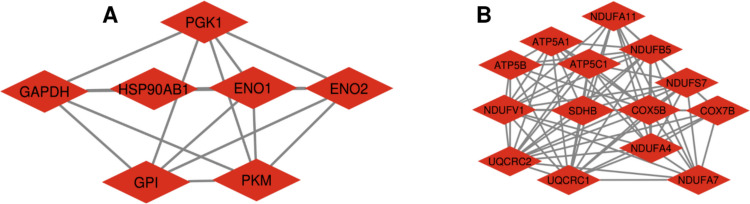
Significant modules identified from the PPI network using the molecular complex detection (MCODE) clustering algorithm. Cluster 1 (**A**) and cluster 2 (**B**) consisting of downregulated DEGs. Red nodes represent downregulated DEGs

A PPI network was constructed to investigate the functional interactions among genes that were significantly differentially expressed between AD brain tissues and non-demented control brain tissues. In the PPI network, which included 259 nodes and 533 interactions (Figure S1), two clusters were identified based on their significance using the MCODE plugin (Fig. 4).

### Gene Ontology and Pathway Enrichment Analysis

Functional enrichment analysis of the four elite genes *COX5B*,* ENO1*,* HSP90AB1*, and *SDHB* revealed strong convergence on biological processes linked to mitochondrial energy metabolism, oxidative phosphorylation, and protein homeostasis, which are central to AD pathophysiology. The GO biological process terms that were primarily enriched reflected an array of cellular processes including cellular respiration, metabolic processing of ATP, oxidative phosphorylation, and responding to oxidative stress, indicating a disrupted coordination between mitochondrial bioenergetic processes and redox homeostasis. The molecular function GO terms that were enriched identified NADH dehydrogenase activity, ATPase binding, and chaperone-mediated protein folding as potential functions that these genes play in maintaining energy transduction and proteostasis. At the cellular component GO term level, there was significant enrichment observed in the mitochondrial inner membrane, the respiratory chain complex I and IV, and the cytosolic chaperone complex, all of which reflect localization of these genes at high-risk subcellular sites subject to AD-associated metabolic stress.

Consistent with this, the KEGG pathway analysis showed that these genes are involved in canonical mitochondrial pathways and neurodegenerative signaling cascades. Specifically, the oxidative phosphorylation and metabolic pathway categories were among the most highly enriched, consistent with the decreased expression of mitochondrial complex subunit genes (*COX5B* and *SDHB*), as has been reported previously in AD transcriptomes. *ENO1*, a key glycolytic enzyme, connected glycolytic flux to neuronal energy demand, suggesting a compensatory metabolic reprogramming under hypometabolic conditions. *HSP90AB1* enrichment within *protein processing in endoplasmic reticulum* and *MAPK signaling pathway* modules indicated potential links between impaired chaperone activity, unfolded protein response, and tau or amyloid misfolding cascades. Together, these functional enrichments delineate a network-level perturbation of mitochondrial respiration, energy production, and proteostasis regulation, hallmarks of AD neuropathology supporting the hypothesis that coordinated suppression of these genes contributes to bioenergetic deficiency, oxidative imbalance, and progressive neurodegeneration in AD.

### In Silico Validations

#### Gene-Tissue Expression

**Fig. 5:**
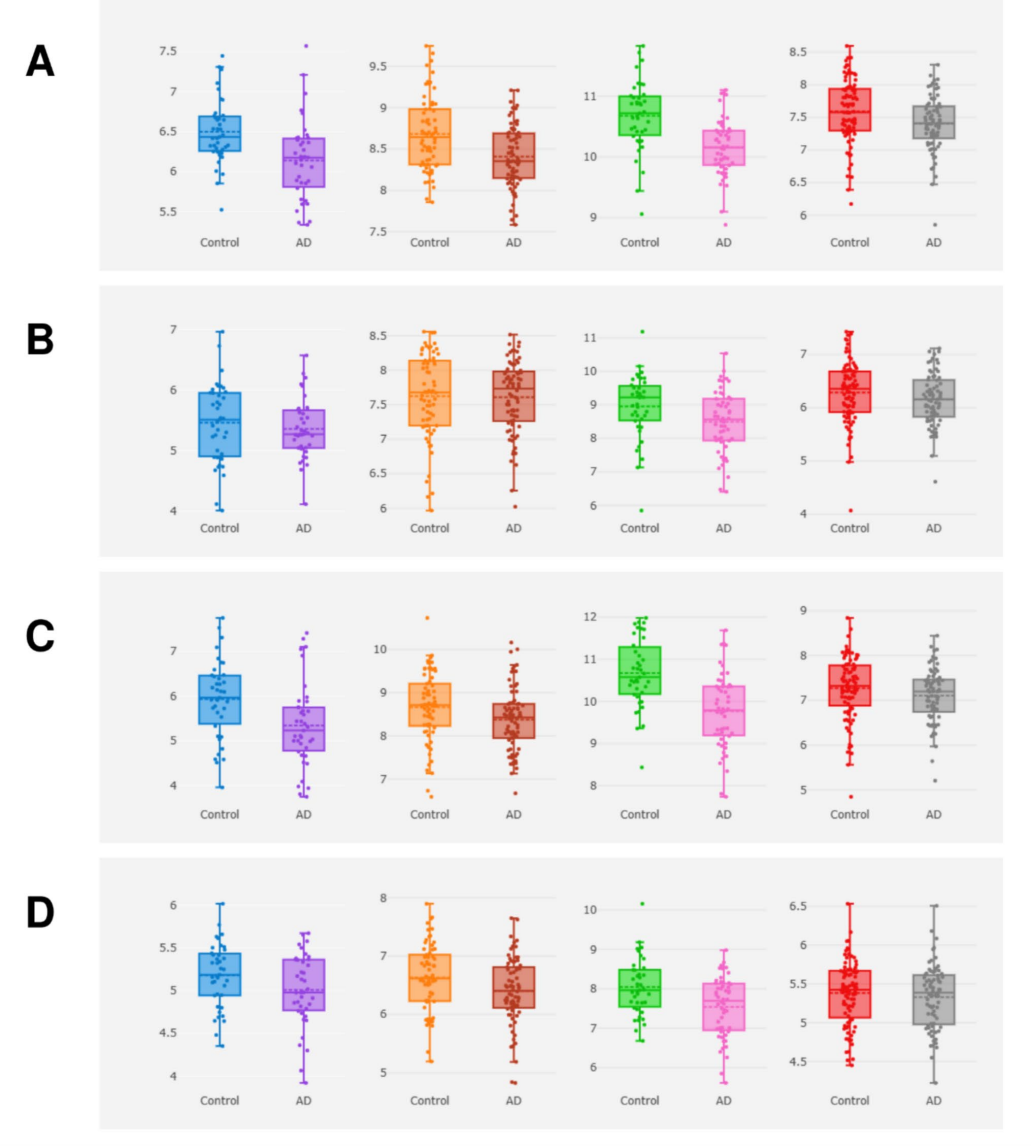
AlzData-based regional expression profiles of the elite genes in AD. Differential expression of **A** *COX5B*, **B** *ENO1*, **C** *HSP90AB1*, and **D** *SDHB* across four AD-relevant brain regions

To externally validate the tissue-level dysregulation of the four elite genes, we queried the AlzData Differential Expression module and compared AD versus non-demented control samples across four AD-relevant brain regions (entorhinal cortex, hippocampus, temporal cortex, and frontal cortex). Consistent with the hub gene directionality observed in the GEO-based analysis, *COX5B* showed a robust and broadly concordant downregulation across all investigated regions, with the strongest decrease in the temporal cortex (log2FC = −0.526, *p* = 1.157e−5, FDR = 4.562e−4), followed by the entorhinal cortex (log2FC = −0.413, *p* = 1.449e−4, FDR = 5.648e−3), hippocampus (log2FC = −0.256, *p* = 5.895e−4, FDR = 0.015), and frontal cortex (log2FC = −0.140, *p* = 0.009, FDR = 0.046) (Fig. 5A; Table S6).

*HSP90AB1* likewise demonstrated marked region-dependent suppression, characterized by a pronounced decrease in the temporal cortex (log2FC = −0.922, *p* = 1.754e−6, FDR = 1.482e−4) and a significant reduction in the entorhinal cortex (log2FC = −0.597, *p* = 4.587e−3, FDR = 0.037), while changes in the hippocampus and frontal cortex were comparatively weaker and did not remain significant after multiple testing correction (Fig. 5C; Table S6). For *SDHB*, downregulation was most evident in the temporal cortex (log2FC = −0.515, *p* = 1.686e−3, FDR = 0.013), whereas the hippocampus showed a nominal decrease and the entorhinal and frontal cortices exhibited smaller, non-significant shifts (Fig. 5D; Table S6). In contrast, *ENO1* displayed limited region-wide alteration, with only a modest reduction in the temporal cortex (log2FC = −0.455, *p* = 0.025) that did not reach FDR significance, and minimal changes in the remaining regions (Fig. 5B; Table S6).

Collectively, the AlzData tissue-level validation supports a consistent, region-biased downregulation pattern that is strongest in the temporal cortex particularly for *COX5B*, *HSP90AB1*, and *SDHB* thereby reinforcing these genes as high-priority candidates linked to AD-relevant molecular vulnerability across affected brain regions (Fig. 5A–D; Table S6).

#### Single-Cell Expression

**Fig. 6:**
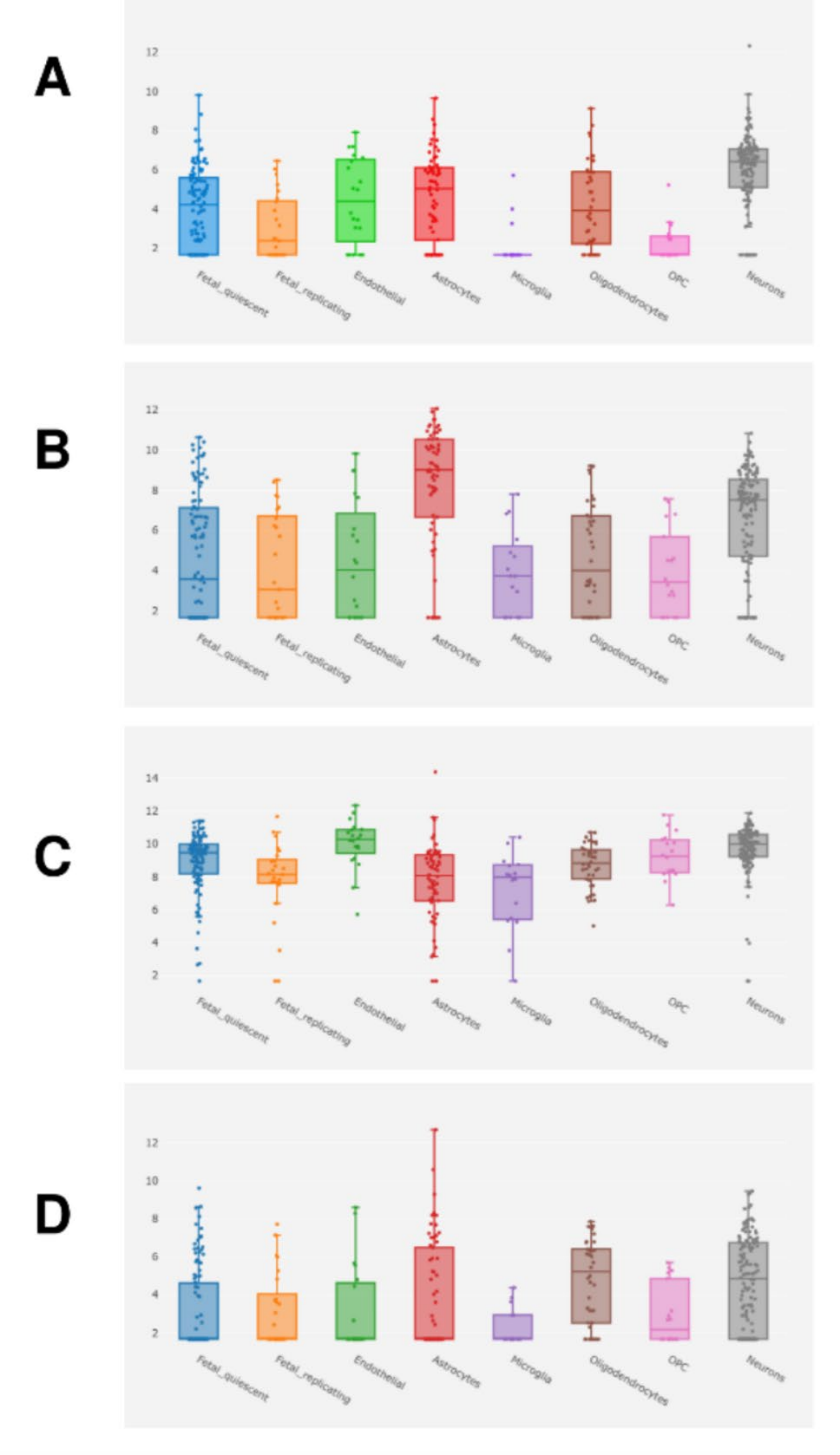
Single-cell expression distribution of the elite genes across major human brain cell types. **A** *COX5B*, **B** *ENO1*, **C** *HSP90AB1*, and **D** *SDHB* expression patterns were retrieved from the AlzData Single Cell Expression module

Single-cell interrogation of the AlzData Single Cell Expression module indicated that the four elite genes exhibit distinct, cell-type stratified expression patterns across major brain cell populations (Fig. 6A–D). *COX5B* displayed comparatively higher expression in neurons and astrocytes, with intermediate levels in endothelial and oligodendrocyte-lineage cells, whereas microglia and OPCs showed the lowest overall signal (Fig. 6A). *ENO1* showed a pronounced enrichment in astrocytes and neurons relative to other populations, consistent with prominent glycolytic activity in these compartments, while microglia and oligodendrocyte-lineage cells exhibited more modest expression ranges (Fig. 6B). In contrast, *HSP90AB1* was broadly expressed across all surveyed cell types, with high median expression in neuronal and non-neuronal lineages, supporting a ubiquitous role for this chaperone in cellular proteostasis in the human brain (Fig. 6C). *SDHB* demonstrated a pattern resembling *COX5B*, with higher expression distributions in astrocytes, oligodendrocytes, and neurons and comparatively reduced levels in microglia and OPCs (Fig. 6D). Collectively, these single-cell profiles suggest that the tissue-level downregulation observed for *COX5B* and *SDHB* in AD is most plausibly driven by neuronal and glial bioenergetic compartments, while the widespread baseline expression of *HSP90AB1* supports its potential contribution to system-wide proteostasis vulnerability in AD.

#### Protein Interaction Analysis

**Fig. 7:**
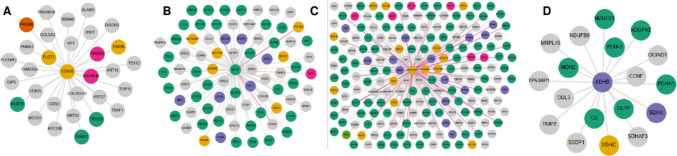
HPA-derived protein–protein interaction networks of the elite genes in AD. Protein interaction maps were exported from the HPA *Interaction* module (protein-class visualization) for **A** COX5B, **B** ENO1, **C** HSP90AB1, and **D** SDHB, with each target protein displayed as the central node and connected to its reported interactors. Node colors indicate protein-class categories: enzymes (green), enzymes/transcription factors (orange), enzymes/transporters (blue), transcription factors (magenta), transcription factors/transporters (light green), transporters (yellow), and other proteins (gray)

To explore the protein-level interaction context of the four elite genes, protein–protein interaction maps were retrieved from the HPA *Interaction* module. The resulting interactomes differed markedly in density and functional composition across targets. The COX5B network was relatively compact (30 interactions) and comprised a limited set of partners spanning regulatory proteins and enzymes, suggesting a focused interaction neighborhood that may reflect constrained connectivity for this mitochondrial electron transport chain component (Fig. 7A). Unlike *ENO1*, the protein–protein interaction network was much larger in number of interactions (78) and comprised a large proportion of enzymes and other metabolic proteins, as well as many glycolytic pathway proteins. These results are consistent with the known central position of *ENO1* in cellular bioenergetic metabolism and the potential for it to connect with a wide variety of metabolic and stress response mechanisms within the brain (Fig. 7B). The largest interactome that we observed was for the *HSP90AB1* protein with 146 interactions. The interactome of *HSP90AB1* formed a highly interconnected protein–protein interaction network that contained an abundance of enzyme class proteins as well as proteins involved in signaling. These characteristics are consistent with the primary function of *HSP90* family members in maintaining proteostasis and in facilitating the stability of a large number of diverse client proteins in many different pathways (Fig. 7C). Finally, SDHB displayed a compact interaction landscape (18 interactions) centered on mitochondrial bioenergetic partners, prominently including succinate dehydrogenase complex components and additional mitochondrial enzymes, supporting its assignment to a tightly organized oxidative phosphorylation/TCA-linked module (Fig. 7D). Collectively, these HPA-derived interaction profiles support mechanistic heterogeneity among the elite genes: COX5B and SDHB align with relatively constrained mitochondrial interaction neighborhoods, ENO1 connects broadly to metabolic enzyme networks, and HSP90AB1 functions as a high-connectivity proteostasis/signaling hub, a feature that is consistent with the mitochondrial dysfunction and proteostasis impairment themes repeatedly implicated in AD pathobiology.

#### Molecular Docking

**Fig. 8:**
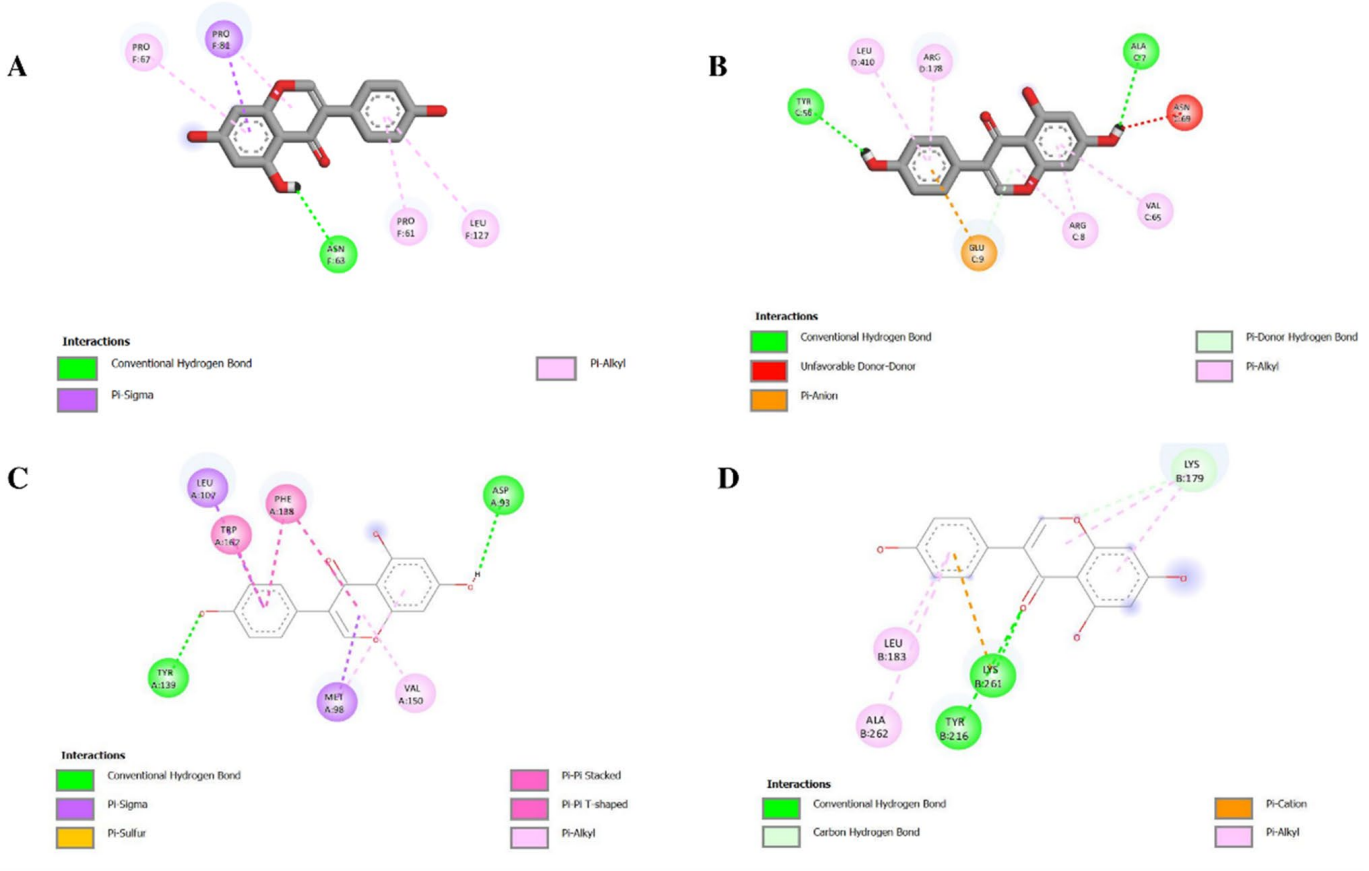
Two-dimensional interaction maps illustrating the molecular docking conformations of genistein with selected protein targets. **A** COX5B, **B** ENO1, **C** HSP90AB1, **D** SDHB

In the COX5B binding pocket, genistein assumes a stabilized and well-oriented structure which is majorly confined by a traditional hydrogen bond with Asn63, a residue that seems to be of significant importance in the stabilization of the ligand. In addition to this polar interaction, other π–alkyl and π–sigma contact with hydrophobic residues such as Pro61, Pro67, Pro81, and Leu127 makes the binding even stronger, as it is supported by aromatic and hydrophobic complementarity (Fig. 8A). The combination of a potent hydrogen bonding capacity and numerous non-covalent interactions indicates that genistein is able to interact with COX5B in an environmentally favorable manner, which is likely to affect its possible modulatory effects on the mitochondrial function.

The interaction between ENO1 and genistein is favorable as the binding energy is predicted to be −7.9 kcal/mol (Table 2), showing that a thermodynamically stable complex is formed. The conventional hydrogen bonds (Tyr56 and Ala7) with the ENO1 binding site stabilize the ligand in its location and allow the correct positioning of the molecule. Further π–alkyl interactions with Val65, Leu410, and Arg178 also increase hydrophobic compatibility, as well as an electrostatic stabilization on the part of a π–anion interaction with Glu9 (Fig. 8B). In spite of unfavorable interaction pattern between donors and the donor (Asn69), the overall interaction pattern indicates a complex binding behavior that could allow genistein to regulate ENO1-related metabolic events.

**Table 2:** Molecular interactions of genistein and resveratrol with COX5B, ENO1, HSP90AB1, and SDHB

Protein	Ligand	Docking score (kcal/mol)
COX5B	Genistein	−7.7
	Resveratrol	−6.5
ENO1	Genistein	−7.9
	Resveratrol	−7.4
HSP90AB1	Genistein	−9.4
	Resveratrol	−9.4
SDHB	Genistein	−7.5
	Resveratrol	−6.5

Genistein exhibited the lowest binding energy among the evaluated targets for HSP90AB1 (− 9.4 kcal/mol) (Table 2), which is representative of a stable ligand protein complex. The standard hydrogen bonds with Asp93 and Tyr139 give anchoring interactions and the binding is further solidified by larger aromatic and hydrophobic interactions. Remarkably, π–π stacked and T-shaped interactions with Phe138 and Trp162 along with π–sigma and π–alkyl interactions involving Leu107, Met98, and Val150 form a richer network of interactions (Fig. 8C). According to this binding architecture, genistein is positioned best to occupy the HSP90AB1 cavity and can modify the chaperone action of this protein.

In the case of SDHB, genistein displayed a comparatively higher binding energy (− 7.5 kcal/mol) (Table 2), indicating a weaker interaction relative to other targets examined in this study. The hydrogen bonds between the conventional ligands and Lys261 and Tyr216 help anchor and orient the ligands, and there is a π–cation bond with Lys261 which adds to the electrostatic stabilization. Further π–alkyl interactions with Leu183 and Ala 262 aid hydrophobic packing in the binding pocket and less strong carbon-hydrogen bond to Lys179 gives minor restraint (Fig. 8D). Taken together, these interactions suggest that SDHB interacts with genistein via an equal mix of polar, electrostatic, and hydrophobic forces, which may have an impact on SDHB-related mitochondrial electron transfer.

**Fig. 9:**
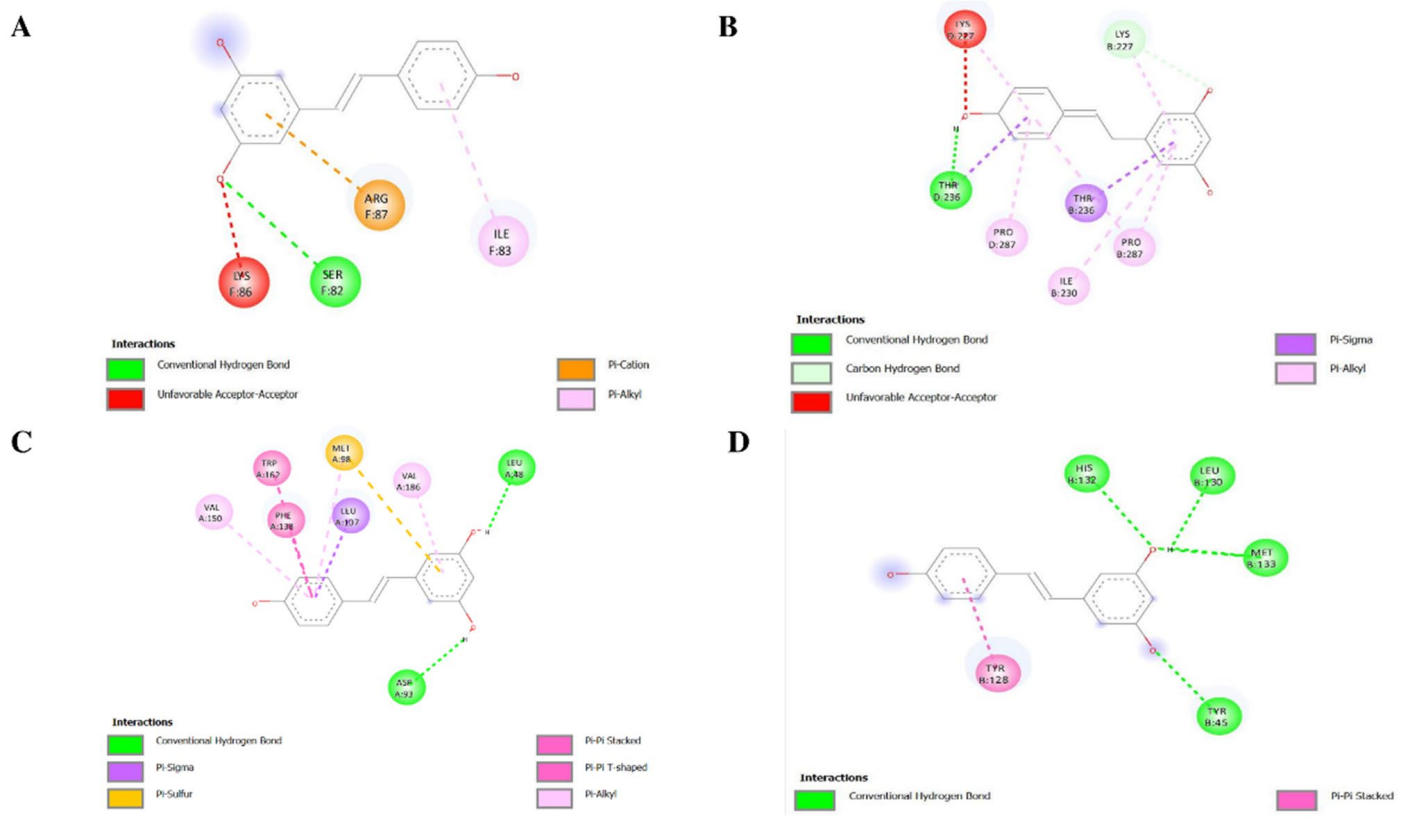
Two-dimensional interaction maps illustrating the molecular docking conformations of resveratrol with selected protein targets. **A** COX5B, **B** ENO1, **C** HSP90AB1, **D** SDHB

In comparison with genistein, resveratrol exhibited a higher binding energy toward COX5B (− 6.5 kcal/mol), indicating a relatively weaker interaction within the same docking framework (Table 2). The binding pose is maintained by a conventional hydrogen bond with Ser82, supported by auxiliary π–cation and π–alkyl interactions involving Arg87 and Ile83, respectively (Fig. 9A). However, the presence of an unfavorable acceptor–acceptor interaction with Lys86 introduces local repulsion, which may compromise overall binding efficiency. All of these characteristics justify the decreased stability of resveratrol-COX5B complex.

Resveratrol exhibited a relatively lower binding energy toward ENO1 (− 7.4 kcal/mol) compared to other evaluated targets, suggesting a potentially favorable interaction within the docking framework (Table 2). A standard hydrogen bond established with Thr236 results in ligand anchoring and π–sigma and π–alkyl interactions between Pro287 and Ile230 are used to promote further stabilization through hydrophobic and aromatic interactions. However, a negative acceptor-acceptor interaction with Lys227 also partially counterbalances these positive interactions indicating poor geometry of binding (Fig. 9B). In spite of this drawback, the interaction profile has provided evidence of a potential role of resveratrol in regulating ENO1-linked glycolytic activity.

Resveratrol exhibited a relatively low predicted binding energy toward HSP90AB1 (− 9.4 kcal/mol), suggesting a potentially favorable interaction. Asp93 and Leu48 firmly anchor the ligand with conventional hydrogen bonds and the complex is further stabilized with extensive aromatic interaction with Phe138 and Trp162 as well as π–alkyl interactions with Leu107, Val150, and Val186. Additionally, a π–sulfur interaction with Met98 contributes to the robustness of the binding mode, suggesting effective accommodation of resveratrol within the HSP90AB1 binding cavity (Fig. 9C).

Lastly, resveratrol showed a predicted binding energy of − 6.5 kcal/mol toward SDHB, indicating a relatively weaker interaction compared to other targets. The docking results revealed the presence of several conventional hydrogen bonds, suggesting potential ligand–protein compatibility. His132, Leu130, Met133, and Tyr45 are some of the residues that play a role in anchoring the ligand to the binding pocket (Fig. 9D). A π–π stacked interaction with Tyr128 further supports aromatic stabilization and proper ligand orientation. Hydrogen bonding dominance and low aromatic contact produce a less compact binding network, which is in agreement with the observed moderate binding affinity, and implies partial regulation of mitochondrial electron transport functions by SDHB.

### Random Forest Classification and Feature Importance

**Fig. 10:**
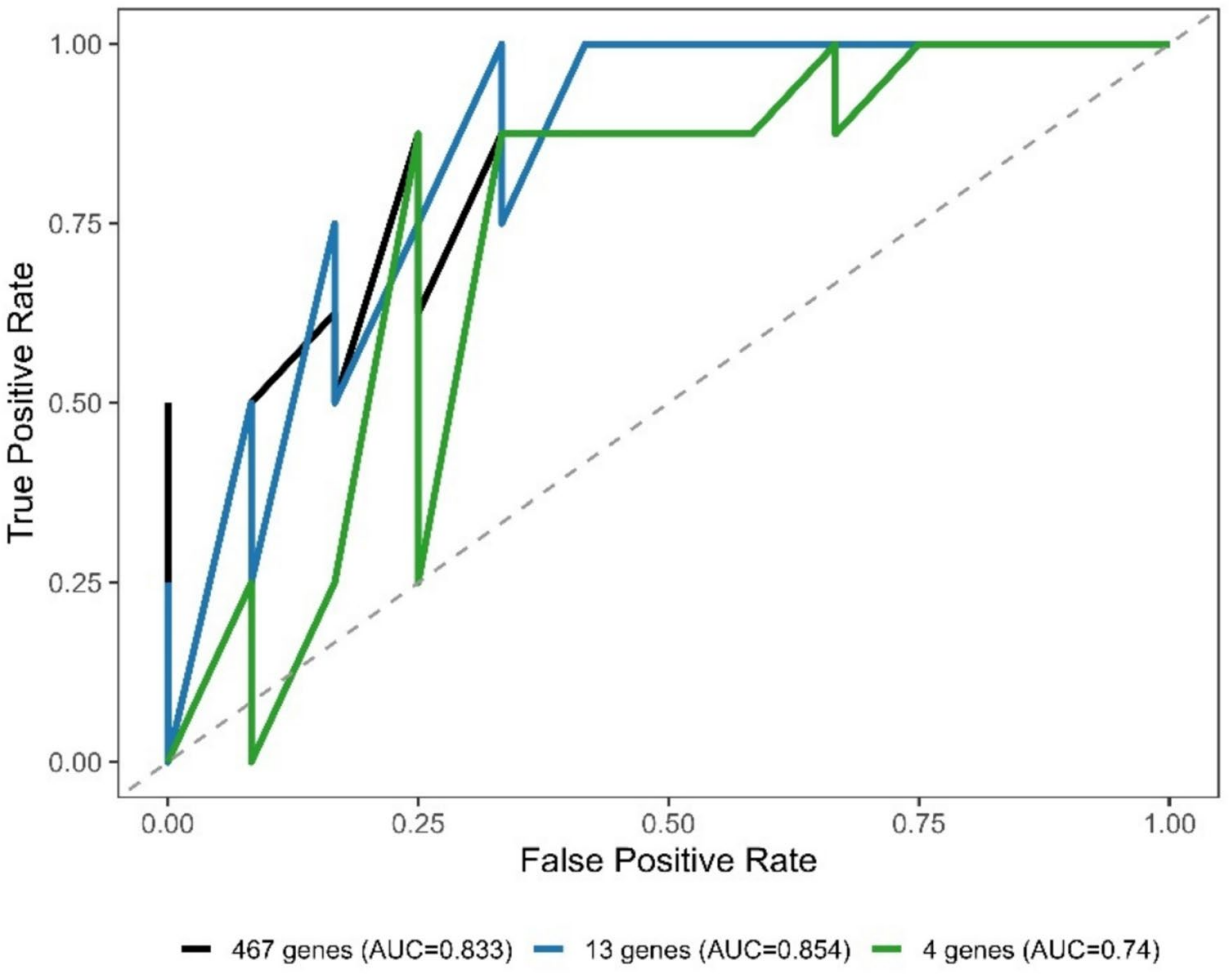
Receiver operating characteristic (ROC) curves comparing the classification performance of random forest models constructed using different gene sets. The model based on 13 hub genes achieved the highest predictive performance (AUC = 0.854), outperforming the full 467-gene model (AUC = 0.833), while the minimal four-gene model retained reasonable discriminative ability (AUC = 0.740)

To assess the predictive performance of the identified gene signatures, random forest models were constructed using different feature sets and evaluated within a stratified training–testing framework. The full model based on 467 shared DEGs achieved a high performance during cross-validation, indicating strong discriminative capability within the training data. When evaluated on the independent test set, the model achieved an AUC of 0.833, demonstrating good generalization performance. To investigate whether a reduced feature space could preserve predictive power, a model based on 13 hub genes was constructed. Notably, this model achieved an AUC of 0.854 on the test set, outperforming the full 467-gene model. This finding suggests that the core predictive signal is concentrated within a smaller subset of biologically relevant genes. Furthermore, a minimal model incorporating four elite genes (COX5B, ENO1, HSP90AB1, and SDHB) retained a reasonable classification performance, achieving an AUC of 0.740 (Fig. 10). Despite the substantial reduction in dimensionality, this model maintained a meaningful discriminative capacity, highlighting the potential of these genes as compact biomarkers. Overall, these results indicate that model performance is not solely driven by the high-dimensional feature space, but rather by a biologically meaningful subset of genes. The improved performance observed in the 13-gene model further supports the robustness and relevance of the hub genes identified through network-based analysis.

## Discussion

AD is the most common form of dementia and a progressive neurodegenerative disorder characterized by hallmark pathologies including amyloid-β deposition, tau hyperphosphorylation, synaptic failure, oxidative stress, mitochondrial dysfunction, and widespread metabolic derangements (D'Alessandro et al. 40. In recent years, a number of mitochondria-associated pathways have been identified as primary factors in AD pathogenesis through the disruption of energy production, the functioning of the electron transport chain and redox balance in both human brain tissue and experimental models [41]. However, despite numerous studies spanning many decades, effective disease-modifying treatments for AD are still lacking in part due to a lack of understanding regarding the molecular links between these disruptions and increased neuronal vulnerability [4]. As such, our studies used an integrated approach including transcriptomics and network analysis across several AD-relevant brain regions to identify elite genes involved in mitochondrial bioenergetics and proteostasis regulation as *COX5B*, *ENO1*, *HSP90AB1*, and *SDHB*. We then validated the expression of these elite genes by analyzing data from the AlzData platform to confirm consistent downregulation of each gene across all brain regions. Additionally, we were able to further characterize their cellular specificity and functional significance at both the single-cell level and protein–protein interaction levels which further supports their utility as mechanistically informative and pharmacologically actionable nodes.

*COX5B* encodes a nuclear-encoded subunit of cytochrome c oxidase (complex IV), the terminal enzyme of the mitochondrial electron transport chain that catalyzes the reduction of oxygen to water and is essential for ATP synthesis via oxidative phosphorylation [42]. Complex IV activity has been repeatedly documented to decline in AD patient brains and biofluids, with meta-analyses demonstrating a significant reduction in cytochrome c oxidase activity in postmortem specimens [43]. These findings implicate impaired electron transport chain function and increased reactive oxygen species generation as contributors to disease progression [44]. *COX5B* and related nuclear-encoded subunits are specifically downregulated in several AD settings, suggesting that compromised assembly or regulation of complex IV may underlie bioenergetic insufficiencies that precede overt neurodegeneration [45]. In our analyses, *COX5B* emerged as one of the most consistently downregulated genes across entorhinal, temporal, frontal, and hippocampal regions, with the greatest repression in the temporal cortex. Protein interaction mapping revealed a focused mitochondrial interactor network, and single-cell profiles underscored neuronal and astrocytic enrichment. Collectively, these results corroborate the concept that *COX5B* suppression contributes to mitochondrial bioenergetic collapse and may serve as a molecular link between electron transport dysfunction and synaptic failure in AD.

*ENO1* encodes α-enolase, a glycolytic enzyme that catalyzes the interconversion of 2-phosphoglycerate to phosphoenolpyruvate, and also participates in stress responses and plasminogen binding [46]. Metabolic hypofunction and glucose hypometabolism are early and consistent features of AD, observed by imaging and postmortem studies, reflecting compromised glycolytic flux and neuronal energy deficits [47]. Oxidative modifications and reduced expression of glycolytic enzymes, including *ENO1*, have been reported in AD cortex and hippocampus, consistent with a systemic metabolic failure that coexists with mitochondrial dysfunction [48]. Moreover, *ENO1* has been shown to interact with Aβ peptides, attenuating their fibrillization and suggesting a potential role in modulating amyloid aggregation [49]. Our multi-regional transcriptomic studies demonstrated that there was a statistically significant though mild *ENO1* repression in AD tissue; this repression occurred across multiple important brain areas as validated by our single-cell RNA-seq data and *ENO1* is predominantly expressed in neurons and astrocytes. We also identified a large number of metabolic interaction partners for *ENO1* through interactome analyses, supporting the notion that *ENO1* repression represents an additional contribution to the metabolic/amyloidogenic disturbances seen in AD.

*HSP90AB1* encodes the constitutive cytosolic isoform of the 90-kDa heat shock protein (*Hsp90β*), a central molecular chaperone involved in protein folding, stabilization, and quality control. Disruption of proteostasis is a core feature of AD, with evidence indicating that chaperone systems become overwhelmed or dysregulated in the presence of aggregated proteins such as tau and Aβ [50]. Members of the Hsp90 family have been implicated in the modulation of tau phosphorylation and aggregation, and reduced levels of Hsp90 paralogs have been reported in AD brain tissue [51]. Together, these observations suggest that compromised chaperone capacity may facilitate proteopathic cascades associated with disease progression [52]. Our data confirmed significant downregulation of *HSP90AB1* across all examined AD brain regions, particularly in entorhinal and temporal cortices. Extensive interactions with enzyme class and signaling proteins reflect its multifaceted role in proteostasis and stress response networks. Evidence for this view is provided by the decreased levels of *HSP90AB1* being associated with the loss of protein folding homeostasis and enhanced susceptibility to tau pathology, and the reduced capacity to respond to stress; all are critical factors contributing to the breakdown of cellular stress resistance. This supports the role of *HSP90AB1* in the proteostasis failure seen in AD.

*SDHB* encodes the iron-sulfur subunit of succinate dehydrogenase (complex II), a dual-function enzyme that participates in the tricarboxylic acid cycle and the electron transport chain. Complex II dysfunction, like that of complex IV, has been implicated in AD via its influence on ATP synthesis efficiency and reactive oxygen species production [53]. Reduced *SDHB* expression and activity contribute to mitochondrial inefficiency and may exacerbate oxidative stress, creating conditions conducive to neuronal injury. In our study, *SDHB* expression was significantly decreased in all examined regions, with the most pronounced effect in the temporal cortex. Protein interaction analysis affirmed its centrality within complex II modules, aligning with prior reports that implicate OXPHOS deficits in AD pathobiology. Evidence supporting these views is that *SDHB* links bioenergetically induced oxidative damage to oxidative pathology and provides additional evidence for the role of integrated mitochondrial dysfunction in the disease. Interestingly, while ENO1 demonstrated direct support in disease-gene association databases such as DisGeNET and MalaCards, COX5B, HSP90AB1, and SDHB exhibited comparatively limited disease-specific annotation. This may reflect the underrepresentation of mitochondrial and proteostasis-associated genes in current AD-centered disease-gene databases despite substantial mechanistic evidence linking these pathways to AD pathology.

In the present study, machine learning analysis was implemented not as a standalone predictive tool but as an integrative validation framework to assess the discriminative capacity of biologically informed gene sets. Importantly, in contrast to conventional approaches relying solely on internal validation metrics, model performance was evaluated using a stratified training–testing framework, thereby providing a more realistic estimate of generalizability. The random forest model constructed using the full set of 467 shared differentially expressed genes achieved strong performance during cross-validation, while yielding a more moderate yet robust performance on the independent test set (AUC = 0.833). This discrepancy suggests a degree of overfitting inherent to high-dimensional feature spaces, a well-recognized limitation in transcriptomic machine learning applications. Notably, dimensionality reduction through biologically guided feature selection resulted in improved model performance. The 13-gene model derived from hub gene analysis outperformed the full model (AUC = 0.854), indicating that the core discriminative signal is concentrated within a smaller subset of functionally relevant genes. This finding underscores the importance of network-based prioritization strategies in enhancing model robustness while mitigating overfitting. Furthermore, the minimal model based on four elite genes (COX5B, ENO1, HSP90AB1, and SDHB) retained a reasonable classification performance (AUC = 0.740), demonstrating that a compact gene panel can preserve substantial predictive information. This result highlights the potential translational relevance of these genes as candidate biomarkers for AD. At the individual gene level, SDHB emerged as the most informative contributor, consistent with its central role in mitochondrial complex II and cellular bioenergetics. Its prominence within the model aligns with extensive evidence linking mitochondrial dysfunction and impaired oxidative phosphorylation to AD pathology. COX5B and ENO1 exhibited moderate contributions, suggesting that their discriminative value arises primarily through synergistic interactions within the multigene network rather than strong individual effects. In contrast, HSP90AB1 showed a comparatively lower contribution, indicating a more supportive or context-dependent role within the predictive framework. The PPI network and cluster-based analyses provided a systems-level framework to interpret these gene-level findings within functional interaction modules, enabling the prioritization of hub genes based on network topology and supporting their biological relevance in AD-related molecular pathways. This finding does not undermine its biological relevance but rather suggests a regulatory or buffering role within the molecular network. As a molecular chaperone involved in protein folding and stress response, HSP90AB1 may exert indirect effects that are not optimally captured by feature importance metrics but remain critical for maintaining proteostasis under neurodegenerative stress conditions.

Collectively this data provides a disease-causing mechanism in which both metabolically induced cell death (*ENO1*,* COX5B*,* SDHB*) and loss of protein quality control (*HSP90AB1*), create an environment of greater susceptibility to disease in AD-affected networks. All of these genes have been found to be downregulated in multiple independent studies and have supporting functional evidence from the scientific literature and in silico analysis. Therefore, it can be suggested that all of these genes may serve as a mechanistic rationale and a target for a multi-modal pharmacologic treatment strategy for AD.

## Conclusion

In conclusion, this study integrates multi-region transcriptomic analysis, network-based prioritization, compound–gene intersection, and molecular docking to identify *COX5B*,* ENO1*,* HSP90AB1*, and *SDHB* as key downregulated genes associated with mitochondrial dysfunction and proteostasis failure in AD. The consistent suppression of these genes across multiple AD-relevant brain regions, together with their central positioning within protein interaction networks, underscores their relevance to core disease mechanisms involving impaired energy metabolism and protein quality control. Importantly, in silico docking analyses revealed stable and energetically favorable interactions of genistein and resveratrol with all four targets, particularly highlighting strong binding profiles for *HSP90AB1* and *ENO1*, thereby supporting the pharmacological tractability of these proteins. While docking results do not establish causality or efficacy, they provide structural support for potential compound-target compatibility within AD-associated molecular pathways. Overall, our findings suggest that coordinated modulation of mitochondrial bioenergetics and proteostasis through multi-target phytochemical interventions represents a rational and biologically grounded strategy for future AD research. These results provide a testable framework for subsequent experimental validation and may inform the development of combinatorial or pleiotropic therapeutic approaches aimed at mitigating neurodegenerative vulnerability in AD.

## Limitations

Several limitations of the present study should be acknowledged. The bioinformatic analyses relied on publicly available transcriptomic datasets, which may introduce unavoidable heterogeneity due to differences in sample sources, data processing pipelines, and patient-specific characteristics. Although standardized analytical approaches were applied to minimize technical variability, such heterogeneity may still influence the reproducibility and generalizability of the findings. A key limitation of the present study is the reliance on a single transcriptomic dataset (GSE5281). This dataset was specifically selected because it uniquely includes multiple AD-relevant brain regions (entorhinal cortex, frontal cortex, hippocampus, and temporal cortex) within a unified experimental framework, enabling direct cross-regional comparisons while minimizing batch effects. However, the use of a single cohort may limit the generalizability of the bioinformatic findings require experimental validation, and the identified differentially expressed genes may still be influenced by dataset-specific variability. Future studies incorporating independent validation cohorts or RNA-seq-based datasets, as well as cross-study integration approaches, would be valuable to further confirm the robustness and reproducibility of these results. The network-based analyses, including PPI networks, primarily reflect associative rather than causal relationships. While these approaches are valuable for identifying functionally connected gene modules and prioritizing candidate hub genes, they do not establish direct mechanistic or causal roles in disease pathogenesis. Therefore, further experimental and functional validation studies are required to elucidate the precise biological roles of these genes in AD. Moreover, although molecular docking provides preliminary in silico insights into ligand–protein interactions, it does not constitute direct evidence of binding affinity or biological activity. The absence of complementary experimental validation (e.g., biochemical binding assays or functional studies) therefore limits the definitive interpretation of these findings. Accordingly, the docking results should be considered exploratory and require further validation in disease-relevant experimental systems. Another limitation is the reliance on CTD-derived compound–gene interactions, which include both experimentally validated and inferred associations and may therefore introduce uncertainty regarding the directness of these relationships. Additionally, the lack of adjustment for potential confounding variables such as age, sex, and APOE genotype represents a limitation of this study, mainly due to incomplete metadata in the utilized dataset. Furthermore, although emerging network medicine approaches such as network proximity analysis may provide additional quantitative insights into drug–disease relationships, such frameworks were beyond the scope of the present study and may represent an important direction for future investigations.

## Supplementary Information

Below is the link to the electronic supplementary material.

ESM 1 (DOCX 738 KB)

## Data Availability

All data used in this study are available in the GEO repository. Data are available from the corresponding author upon reasonable request.
