# Supplementary material for: Integrative Transcriptomic, Network, and Machine Learning Analyses Identify Genistein and Resveratrol-Associated Therapeutic Targets in Alzheimer’s Disease
Source: Mol Neurobiol. 2026 Jun 2;63(1):666. doi: 10.1007/s12035-026-05973-y (PMC13230253; doi:10.1007/s12035-026-05973-y)
Supplement: Supplementary file 1 — (DOCX 738 KB) [file 12035_2026_5973_MOESM1_ESM.docx]

**Table S1.** List of differentially expressed genes (DEGs) distributions from each brain region. GEO; gene expression omnibus.

| GSE5281 | GEO | Total of DEGs | Upregulated DEGs | Downregulated DEGs |
| --- | --- | --- | --- | --- |
|  | Entorhinal Cortex | 5664 | 2557 | 3107 |
|  | Frontal Cortex | 6888 | 481 | 6407 |
|  | Hippocampus | 5189 | 3361 | 1828 |
|  | Temporal Cortex | 8766 | 2477 | 6289 |

**Table S2.** Shared up-regulated and down-regulated DEGs in GEOs derived from AD brain tissues and adjacent healty tissues. AD; Alzheimer Disease, DEGs; differentially expressed genes, GEO; gene expression omnibus.

| Shared up-regulated (*p*-value <0.05, \|log2FC\| ≥ 1) genes in AD datasets | | | | |
| --- | --- | --- | --- | --- |
| *ACTA2* | *EZR* | *MEG3* | *PPP1R3D* | *TFEB* |
| *ADGRL4* | *FBLN1* | *MKLN1* | *PRKD3* | *TGFBR3* |
| *ANKRD36* | *FBXO32* | *MYH11* | *PTAR1* | *TNFRSF10B* |
| *ANP32B* | *FOXC1* | *MYO10* | *PTMA* | *TNRC6C* |
| *ARHGEF10* | *GLIS3* | *NFIA* | *PTPN2* | *TOB2* |
| *ARHGEF40* | *GOLIM4* | *NFX1* | *PXDC1* | *TRAK1* |
| *ATP8B1* | *GPAM* | *NKTR* | *QKI* | *TRIM14* |
| *BBX* | *HDAC7* | *NOTCH2* | *RASEF* | *TRIM56* |
| *CFLAR* | *HIPK2* | *NSUN6* | *RBM33* | *UBE2I* |
| *COL1A1* | *IFI16* | *NTRK2* | *RGS12* | *UBE3A* |
| *COL27A1* | *IL6ST* | *NUCKS1* | *RHOJ* | *WDR1* |
| *CPM* | *ITGB8* | *PALLD* | *RHOQ* | *WNK1* |
| *CSNK1A1* | *ITPKB* | *PCSK5* | *RRBP1* | *WWC1* |
| *CXCR4* | *ITPR2* | *PECAM1* | *RUFY3* | *WWTR1* |
| *CYYR1* | *KCNE4* | *PMP2* | *SCD* | *YBX3* |
| *DCN* | *KCNN3* | *PNISR* | *SEC22C* | *ZBED6* |
| *DGKG* | *LATS2* | *POLH* | *SLC25A29* | *ZBTB20* |
| *DKFZP586B0319* | *LIFR* | *PON2* | *SOX9* | *ZBTB7A* |
| *DNASE1* | *LUC7L3* | *POU3F2* | *SPPL3* | *ZC3H7B* |
| *DTNA* | *MAFF* | *PPARA* | *SSPN* | *ZDHHC21* |
| *EML3* | *MALAT1* | *PPFIBP1* | *TBL1X* | *ZFAND6* |
| *ERBB4* | *MAP4K4* | *PPP1R12B* | *TFAP2C* | *ZFHX3* |
| *ZFP36L1* | *ZNF366* | *ZSCAN30* |  |  |
| Shared down-regulated (*p*-value <0.05, \|log2FC\| ≤ 1) genes in AD datasets | | | | |
| *AAGAB* | *CIRBP* | *HHLA2* | *NDUFA7* | *SDR16C5* |
| *AARS* | *CKAP5* | *HIKESHI* | *NDUFAF3* | *SEC14L2* |
| *ABCF2* | *CLEC2L* | *HMGCR* | *NDUFB5* | *SEPHS1* |
| *ABHD2* | *CLPP* | *HMGN4* | *NDUFS7* | *SGIP1* |
| *ACLY* | *CLTB* | *HNRNPL* | *NDUFV1* | *SKP1* |
| *ACOT7* | *CNOT10* | *HS6ST3* | *NHP2* | *SLC19A1* |
| *ACTB* | *COMMD4* | *HSP90AB1* | *NIPAL3* | *SLC25A11* |
| *ACTN2* | *COPS8* | *ICA1* | *NMRAL1* | *SLC25A14* |
| *ACTR1A* | *COX5B* | *IDH3B* | *NPM1* | *SLC27A4* |
| *ADAM23* | *COX7B* | *IDH3G* | *NRXN3* | *SLC27A5* |
| *ADARB1* | *CRYBA2* | *IMPDH2* | *NSDHL* | *SLC35F3* |
| *ADD2* | *CSNK2B* | *INA* | *NSG1* | *SLC9A6* |
| *AGAP2* | *CSTF1* | *ITFG1* | *NTPCR* | *SLIRP* |
| *AKIRIN2* | *CUL3* | *ITGB1* | *NUDT2* | *SLIT3* |
| *ALKBH7* | *CYB561* | *KARS* | *OAZ1* | *SMARCA4* |
| *AMPH* | *CYB5A* | *KAT5* | *OCIAD1* | *SNAP25* |
| *ANAPC5* | *CYFIP2* | *KCNAB2* | *OXLD1* | *SNCA* |
| *ANKRD36BP2* | *DHDDS* | *KCNS2* | *P4HTM* | *SPATA7* |
| *ANXA6* | *DHRS7B* | *KIFAP3* | *PACSIN1* | *SPIN3* |
| *AP1M1* | *DHX30* | *KIZ* | *PARPBP* | *SQLE* |
| *AP2A2* | *DKK3* | *KLC1* | *PARVA* | *SRA1* |
| *AP2S1* | *DMAP1* | *LAMB1* | *PCMT1* | *SRPK2* |
| *AP3M2* | *DMD* | *LARGE1* | *PCYOX1L* | *SRSF3* |
| *ARHGEF9* | *DNAAF1* | *LARP1B* | *PDK3* | *SSU72* |
| *ARL6* | *DNAH1* | *LARP4B* | *PEX13* | *STMN2* |
| *ARMCX4* | *DNM3* | *LDLR* | *PGK1* | *STX8* |
| *ARPC4* | *DNPH1* | *LGALS8* | *PKM* | *SUB1* |
| *ARPP19* | *DNTTIP1* | *LMBR1* | *PLD3* | *SUPT4H1* |
| *ARSG* | *DUS4L* | *LMLN* | *PNN* | *SUSD4* |
| *ASAH1* | *DYNC1H1* | *LMO3* | *POLDIP2* | *SV2B* |
| *ASB1* | *DZIP3* | *LMO4* | *PPIH* | *SYCE1* |
| *ASMTL* | *EBP* | *LRPAP1* | *PPP1R7* | *SYN2* |
| *ATG2B* | *EEF1A2* | *LSM3* | *PPP2R2D* | *TCEA2* |
| *ATP1A1* | *EGR1* | *LSM4* | *PQBP1* | *TCF3* |
| *ATP2B3* | *EIF2B3* | *LUZP1* | *PRDM2* | *TDRKH* |
| *ATP5A1* | *EIF3K* | *LYPD8* | *PREP* | *TIMM17A* |
| *ATP5B* | *EMC4* | *MACROD2* | *PROSC* | *TIMM50* |
| *ATP5C1* | *ENO1* | *MAGED1* | *PRR3* | *TM7SF2* |
| *ATP6V0B* | *ENO2* | *MAP2K5* | *PSMA5* | *TMEFF2* |
| *ATP6V1A* | *ENOSF1* | *MAPK11* | *PSMB2* | *TMEM135* |
| *ATP6V1E1* | *EPB41L3* | *MAPRE3* | *PSMB3* | *TMEM191A* |
| *ATP6V1G2* | *EPB41L4B* | *MAPT* | *PSMB4* | *TMEM232* |
| *ATP6V1H* | *ERICH1* | *MBP* | *PSMB7* | *TMEM38A* |
| *B3GALNT1* | *EXOSC4* | *MDH1* | *PSMC2* | *TMEM59* |
| *B9D1* | *FABP3* | *ME3* | *PSMD1* | *TMEM97* |
| *BRWD1* | *FAHD2A* | *MED23* | *PTP4A1* | *TMOD1* |
| *CA10* | *FAM149B1* | *MED6* | *PTPN3* | *TOLLIP* |
| *CABP1* | *FAM188A* | *MEF2C* | *PTPRR* | *TPD52* |
| *CADM4* | *FAM86B3P* | *METTL3* | *RABEP1* | *TPM1* |
| *CADPS* | *FARSA* | *MFSD4A* | *RAD1* | *TRIM2* |
| *CALM1* | *FDFT1* | *MFSD4B* | *RAD51C* | *TRIM36* |
| *CALY* | *FHL1* | *MIF* | *RAP1GDS1* | *TSPAN3* |
| *CAMK4* | *GABBR2* | *MKKS* | *RBFOX1* | *TUBA4A* |
| *CAPNS1* | *GABRA5* | *MOCS2* | *RBP4* | *TUBB* |
| *CAPRIN2* | *GABRD* | *MPV17* | *REEP1* | *TUBB3* |
| *CCAR2* | *GAPDH* | *MRPL30* | *RFWD3* | *TUBB4B* |
| *CCK* | *GDE1* | *MRPL37* | *RFX5* | *TUSC3* |
| *CCT5* | *GGCX* | *MRPL4* | *RGS7* | *TXN* |
| *CCT7* | *GHITM* | *MRPS15* | *RNF25* | *UBE2F* |
| *CD2BP2* | *GLS* | *MRPS23* | *ROMO1* | *UBE2N* |
| *CDC37* | *GLS2* | *MRTO4* | *RRAGA* | *UBE2QL1* |
| *CDC42* | *GNAS* | *MTPAP* | *RRAGB* | *UCHL1* |
| *CDK7* | *GOSR2* | *MTSS1* | *RTN1* | *UQCRC1* |
| *CEP85* | *GOT1* | *NAP1L5* | *RTN4IP1* | *UQCRC2* |
| *CFAP52* | *GPI* | *NAPA* | *RUVBL1* | *UTP4* |
| *CFAP69* | *GRIA1* | *NAV3* | *SAMM50* | *VPS53* |
| *CHAF1B* | *GRIN1* | *NCAPH2* | *SAP18* | *WARS* |
| *CHCHD6* | *GRM8* | *NDRG3* | *SARS* | *WDR46* |
| *CHGB* | *HBS1L* | *NDUFA11* | *SCCPDH* | *YKT6* |
| *CHRM3* | *HDLBP* | *NDUFA4* | *SDHB* | *YWHAZ* |
| *ZC3H14* | *ZC4H2* | *ZDHHC4* | *ZNF415* |  |

**Table S3.** Top 15 genes evaluated in the PPI network using three calculation methods and employing CytoHubba in Cytoscape.

The overlap hub genes in the top 15 by three ranked methods respectively in cytoHubba are highlighted in bold.

| **MNC** | **Degree** | **EPC** |
| --- | --- | --- |
| \| *MAPT* \| \| --- \| \| *NDUFA7* \| \| *ATP5C1* \| \| *ATP5A1* \| \| *NDUFV1* \| \| ***SDHB*** \| \| ***COX5B*** \| \| ***HSP90AB1*** \| \| \| *NDUFB5* \| \| *UQCRC1* \| \| *ATP5B* \| \| *UQCRC2* \| \| *NDUFS7* \| \| ***ENO1*** \| \| *NDUFA4* \| | \| *ACTB* \| \| --- \| \| *GAPDH* \| \| *ATP5C1* \| \| *ATP5A1* \| \| *NDUFV1* \| \| ***SDHB*** \| \| ***HSP90AB1*** \| \| *UQCRC1* \| \| ***COX5B*** \| \| *ATP5B* \| \| *NDUFB5* \| \| *UQCRC2* \| \| *NDUFS7* \| \| ***ENO1*** \| \| *NDUFA4* \| | \| *GAPDH* \| \| --- \| \| *ATP5A1* \| \| *ATP5C1* \| \| *ATP5B* \| \| *ACTB* \| \| *NDUFV1* \| \| *UQCRC1* \| \| *NDUFB5* \| \| *UQCRC2* \| \| ***COX5B*** \| \| ***SDHB*** \| \| ***ENO1*** \| \| ***HSP90AB1*** \| \| \| *NDUFA4* \| \| *NDUFS7* \| |

**Table S4.** The overlap targ*et genes between 2 chemical compounds (Genistein and Resveratrol).*

| \| *ABCA1* \| *CPN1* \| *HES1* \| *NASP* \| *SEC1* \| \| --- \| --- \| --- \| --- \| --- \| \| *ABCA6* \| *CPNE7* \| *HGF* \| *NBN* \| *SEL1L* \| \| *ABCB1* \| *CPT1A* \| *HHIP* \| *NCAPD2* \| *SELENBP1* \| \| *ABCB1B* \| *CRB2* \| *HID1* \| *NCAPG* \| *SELENOV* \| \| *ABCC1* \| *CREB1* \| *HIF1A* \| *NCMAP* \| *SELL* \| \| *ABCC2* \| *CREB3L3* \| *HIVEP1* \| *NCOA1* \| *SELP* \| \| *ABCC3* \| *CREB3L4* \| *HK2* \| *NCOA2* \| *SEMA3D* \| \| *ABCC5* \| *CRHR1* \| *HMCN2* \| *NCOA3* \| *SERHL* \| \| *ABCC8* \| *CRHR2* \| *HMGA2* \| *NCOA4* \| *SERPINA1* \| \| *ABCC9* \| *CRIP1* \| *HMGB2* \| *NCOA6* \| *SERPINA11* \| \| *ABCG2* \| *CRIP2* \| *HMGCR* \| *NDC80* \| *SERPINA1E* \| \| *ABHD11* \| *CRISPLD2* \| *HMGCS2* \| *NDRG1* \| *SERPINB12* \| \| *ABI1* \| *CRP* \| *HMGN1* \| *NDUFA13* \| *SERPINB2* \| \| *ABL1* \| *CRYAA* \| *HMGN2* \| *NDUFA5* \| *SERPINB5* \| \| *ACAA2* \| *CRYGN* \| *HMOX1* \| *NDUFB1* \| *SERPINE1* \| \| *ACACA* \| *CSF1* \| *HNF1A* \| *NDUFB3* \| *SESN1* \| \| *ACACB* \| *CSF1R* \| *HNRNPA2B1* \| *NDUFS1* \| *SESN2* \| \| *ACAD10* \| *CSF2* \| *HNRNPAB* \| *NDUFS3* \| *SF1* \| \| *ACAD11* \| *CSF3* \| *HNRNPC* \| *NDUFS4* \| *SF3B5* \| \| *ACE* \| *CSNK1G1* \| *HNRNPH1* \| *NECAP2* \| *SFTPD* \| \| *ACHE* \| *CSRP1* \| *HNRNPUL2* \| *NEDD9* \| *SFXN2* \| \| *ACLY* \| *CSRP2* \| *HOMER2* \| *NEFH* \| *SGCB* \| \| *ACNAT2* \| *CSRP3* \| *HOPX* \| *NEIL2* \| *SGK3* \| \| *ACOT4* \| *CST3* \| *HOXA1* \| *NELL2* \| *SGPP2* \| \| *ACOT9* \| *CSTA* \| *HOXA13* \| *NEU4* \| *SH2D4B* \| \| *ACOX1* \| *CTBS* \| *HOXB6* \| *NEUROG1* \| *SHC1* \| \| *ACP3* \| *CTCFLOS* \| *HOXB9* \| *NFAT5* \| *SHC3* \| \| *ACP5* \| *CTH* \| *HP* \| *NFATC1* \| *SHH* \| \| *ACSL3* \| *CTHRC1* \| *HPCA* \| *NFE2L2* \| *SHOX2* \| \| *ACSM3* \| *CTNNB1* \| *HPCAL1* \| *NFE2L2B* \| *SIAH2* \| \| *ACTA1* \| *CTNND2* \| *HPRT1* \| *NFKB1* \| *SIRT1* \| \| *ACTA2* \| *CTPS1* \| *HPSE* \| *NFKB2* \| *SIX4* \| \| *ACTB* \| *CTRB1* \| *HPX* \| *NFKBIA* \| *SKAP2* \| \| *ACTBL2* \| *CTSD* \| *HR* \| *NFKBIZ* \| *SKIL* \| \| *ACTN2* \| *CTSH* \| *HSD11B1* \| *NFS1* \| *SLC10A2* \| \| *ADA* \| *CTSK* \| *HSD11B2* \| *NGF* \| *SLC12A4* \| \| *ADAM9* \| *CUX1* \| *HSD17B1* \| *NGFR* \| *SLC12A5* \| \| *ADAMTS2* \| *CXCL10* \| *HSD17B12* \| *NHP2* \| *SLC15A3* \| \| *ADAMTS4* \| *CXCL12* \| *HSD17B2* \| *NHSL3* \| *SLC16A1* \| \| *ADAMTS7* \| *CXCL14* \| *HSD3B1* \| *NID2* \| *SLC18A2* \| \| *ADCY4* \| *CXCL15* \| *HSD3B5* \| *NKX3-1* \| *SLC19A1* \| \| *ADGRD1* \| *CXCL5* \| *HSP90AA1* \| *NLN* \| *SLC1A2* \| \| *ADGRE5* \| *CXCL8* \| *HSP90AB1* \| *NLRP12* \| *SLC1A3* \| \| *ADIPOR2* \| *CXCR4* \| *HSP90B1* \| *NLRP3* \| *SLC1A4* \| \| *ADM2* \| *CYB561* \| *HSPA12A* \| *NMNAT1* \| *SLC1A5* \| \| *ADRB1* \| *CYB5A* \| *HSPA1A* \| *NMRK1* \| *SLC22A1* \| \| *ADSL* \| *CYB5R3* \| *HSPA1B* \| *NMU* \| *SLC22A2* \| \| *AGER* \| *CYBA* \| *HSPA2* \| *NOC2L* \| *SLC22A6* \| \| *AGR2* \| *CYBB* \| *HSPA4L* \| *NOD1* \| *SLC25A19* \| \| *AGRN* \| *CYCS* \| *HSPA9* \| *NOP2* \| *SLC25A22* \| \| *AGT* \| *CYGB* \| *HSPB1* \| *NOS1AP* \| *SLC27A2* \| \| *AHCYL1* \| *CYP11A1* \| *HSPB7* \| *NOS2* \| *SLC28A2* \| \| *AHNAK* \| *CYP17A1* \| *HSPB8* \| *NOS2A* \| *SLC2A1* \| \| *AHR* \| *CYP19A1* \| *HSPB9* \| *NOS3* \| *SLC2A2* \| \| *AHSG* \| *CYP1A* \| *HSPD1* \| *NOTCH2* \| *SLC2A4* \| \| *AK2* \| *CYP1A1* \| *HSPG2* \| *NPAS3* \| *SLC31A1* \| \| *AKR1C3* \| *CYP1A2* \| *HSPH1* \| *NPC2* \| *SLC34A3* \| \| *AKT1* \| *CYP1B1* \| *HTR1B* \| *NPHS1* \| *SLC35F3* \| \| *AKT2* \| *CYP26B1* \| *HYAL3* \| *NPNT* \| *SLC35F4* \| \| *AKT3* \| *CYP2C19* \| *HYOU1* \| *NPPC* \| *SLC39A8* \| \| *ALB* \| *CYP2C29* \| *ICAM1* \| *NPR1* \| *SLC3A1* \| \| *ALCAM* \| *CYP2C9* \| *ID1* \| *NPW* \| *SLC3A2* \| \| *ALDH1A1* \| *CYP2E1* \| *ID3* \| *NPY1R* \| *SLC44A4* \| \| *ALDH1A2* \| *CYP2R1* \| *ID4* \| *NPY2R* \| *SLC5A1* \| \| *ALDH1A3* \| *CYP3A4* \| *IDI1* \| *NQO1* \| *SLC5A5* \| \| *ALDH1B1* \| *CYP4A12A* \| *IER3* \| *NR0B2* \| *SLC6A14* \| \| *ALDH3A2* \| *CYP4A14* \| *IER5L* \| *NR1D1* \| *SLC6A18* \| \| *ALDOA* \| *CYP4B1* \| *IFIH1* \| *NR1H3* \| *SLC6A2* \| \| *ALDOB* \| *CYP4F2* \| *IFIT3* \| *NR1H4* \| *SLC6A6* \| \| *ALDOC* \| *CYP51* \| *IFITM1* \| *NR1I2* \| *SLC7A11* \| \| *ALG8* \| *CYP7A1* \| *IFITM3* \| *NR2F2* \| *SLC7A2* \| \| *ALOX12* \| *DACT2* \| *IFNA1* \| *NR3C1* \| *SLC7A5* \| \| *ALPI* \| *DAPL1* \| *IFNG* \| *NR4A1* \| *SLC7A7* \| \| *ALPL* \| *DARS1* \| *IFRD2* \| *NR5A1* \| *SLC8A1* \| \| *ALPP* \| *DCLK1* \| *IFT88* \| *NRAP* \| *SLCO1A1* \| \| *AMH* \| *DCN* \| *IGF1* \| *NRAS* \| *SLCO1A4* \| \| *AMIGO2* \| *DCT* \| *IGF1R* \| *NREP* \| *SLCO2A1* \| \| *ANAPC1* \| *DCTN4* \| *IGF2* \| *NRIP1* \| *SLCO4A1* \| \| *ANGPTL4* \| *DCXR* \| *IGF2R* \| *NRXN3* \| *SLFN5* \| \| *ANKRD36B* \| *DDAH2* \| *IGFBP1* \| *NTS* \| *SLK* \| \| *ANKRD53* \| *DDC* \| *IGFBP2* \| *NUDT7* \| *SMAD2* \| \| *ANO1* \| *DDIT3* \| *IGFBP3* \| *NUMA1* \| *SMAD3* \| \| *ANP32A* \| *DDIT4* \| *IGFBP4* \| *NUP155* \| *SMAD7* \| \| *ANXA1* \| *DEGS2* \| *IGFBP5* \| *NUP58* \| *SMARCA4* \| \| *ANXA2* \| *DENND11* \| *IHH* \| *NUPR1* \| *SMARCA5* \| \| *ANXA3* \| *DEPTOR* \| *IKBKB* \| *NXPH1* \| *SMC2* \| \| *ANXA5* \| *DERA* \| *IL10* \| *OARD1* \| *SMC4* \| \| *ANXA6* \| *DERL3* \| *IL12A* \| *OCLN* \| *SMC5* \| \| *APAF1* \| *DFFA* \| *IL12B* \| *OGFR* \| *SMURF1* \| \| *APLP2* \| *DGKE* \| *IL13* \| *OLFM1* \| *SNAI1* \| \| *APOA1* \| *DGUOK* \| *IL17A* \| *OLFML3* \| *SNAI2* \| \| *APOB* \| *DHFR* \| *IL17RB* \| *OTX1* \| *SNHG32* \| \| *APOBEC3B* \| *DHRS2* \| *IL18* \| *OXT* \| *SNRPA* \| \| *APOC1* \| *DHRS3* \| *IL1A* \| *OXTR* \| *SNRPB2* \| \| *APOE* \| *DHRS4* \| *IL1B* \| *P2RX7* \| *SNTA1* \| \| *APOV1* \| *DHX38* \| *IL1R1* \| *P4HA1* \| *SNX30* \| \| *APP* \| *DHX9* \| *IL1RN* \| *P4HA3* \| *SOCS2* \| \| *APPBP2* \| *DIO1* \| *IL2* \| *PADI1* \| *SOCS3* \| \| *APRT* \| *DIO2* \| *IL34* \| *PAICS* \| *SOD1* \| \| *AQP1* \| *DIPK1C* \| *IL36G* \| *PAK1* \| *SOD2* \| \| *AQP3* \| *DLD* \| *IL4* \| *PAK2* \| *SOD3* \| \| *AQP7* \| *DLGAP5* \| *IL6* \| *PAK3* \| *SOX11* \| \| *AR* \| *DLK1* \| *ILF2* \| *PARP1* \| *SOX17* \| \| *ARC* \| *DLL3* \| *ILF3* \| *PAX4* \| *SOX2* \| \| *AREG* \| *DMBT1* \| *IMPDH2* \| *PBK* \| *SOX4* \| \| *ARF1* \| *DMD* \| *INHBA* \| *PCCA* \| *SOX8* \| \| *ARF4* \| *DMKN* \| *INHBB* \| *PCDH10* \| *SOX9* \| \| *ARFGEF3* \| *DNAJB4* \| *INPP4B* \| *PCDH8* \| *SP1* \| \| *ARG1* \| *DNAJB9* \| *INPP5B* \| *PCK1* \| *SP5* \| \| *ARHGDIA* \| *DNAJC16* \| *INS1* \| *PCK2* \| *SP7* \| \| *ARL3* \| *DNAJC21* \| *INSIG1* \| *PCLAF* \| *SPAG5* \| \| *ARMC10* \| *DNAJC3* \| *INTS10* \| *PCNA* \| *SPARC* \| \| *ARMCX3* \| *DNMT1* \| *IPO13* \| *PCP4* \| *SPDEF* \| \| *ARNT* \| *DNMT3A* \| *IPO7* \| *PCSK5* \| *SPMIP8* \| \| *ARPC1B* \| *DNMT3B* \| *IQGAP2* \| *PCSK9* \| *SPP1* \| \| *ASAH1* \| *DOK4* \| *IRAK1* \| *PDCD11* \| *SPPL2C* \| \| *ASB7* \| *DPP6* \| *IRAK2* \| *PDCD4* \| *SPSB3* \| \| *ASH2L* \| *DPY19L3* \| *IRF2BP1* \| *PDCD7* \| *SPTBN1* \| \| *ASNS* \| *DRD1* \| *IRF3* \| *PDE6H* \| *SPTLC2* \| \| *ASPH* \| *DSN1* \| *IRS1* \| *PDGFB* \| *SQLE* \| \| *ASPM* \| *DST* \| *IRS2* \| *PDGFRA* \| *SQSTM1* \| \| *ASRGL1* \| *DSTN* \| *IRX1* \| *PDGFRB* \| *SRC* \| \| *ASS1* \| *DTYMK* \| *ISG20* \| *PDGFRL* \| *SRD5A2* \| \| *ATAD2* \| *DUOX1* \| *ISOC1* \| *PDHA1* \| *SRD5A3* \| \| *ATF1* \| *DUOXA1* \| *ISYNA1* \| *PDIA3* \| *SREBF1* \| \| *ATF2* \| *DUOXA2* \| *ITGA1* \| *PDZK1* \| *SRPRA* \| \| *ATF3* \| *DUSP1* \| *ITGA2* \| *PELI2* \| *SRPRB* \| \| *ATG5* \| *DVL1* \| *ITGA3* \| *PF4* \| *SRSF1* \| \| *ATM* \| *DYNC2I1* \| *ITGA4* \| *PFKFB3* \| *SSR1* \| \| *ATP1A1* \| *DYRK2* \| *ITGA5* \| *PFKP* \| *SSX2IP* \| \| *ATP1B1* \| *E2F2* \| *ITGAM* \| *PGF* \| *ST8SIA4* \| \| *ATP1B3* \| *E2F7* \| *ITGAV* \| *PGK1* \| *STAR* \| \| *ATP2A2* \| *EBP* \| *ITGB1* \| *PGPEP1* \| *STARD3* \| \| *ATP2A3* \| *ECE1* \| *ITGB3* \| *PGR* \| *STAT1* \| \| *ATP4B* \| *ECH1* \| *ITGB3BP* \| *PGRMC1* \| *STAT3* \| \| *ATP5F1E* \| *ECT2* \| *ITGBL1* \| *PHF14* \| *STAT4* \| \| *ATP5MC1* \| *EDARADD* \| *ITPA* \| *PHF20L1* \| *STAT5A* \| \| *ATP5MC2* \| *EDC4* \| *ITPR1* \| *PHF24* \| *STBD1* \| \| *ATP5PO* \| *EDN1* \| *ITPR3* \| *PHLDA2* \| *STEAP4* \| \| *ATP6V0A4* \| *EDN2* \| *JAK1* \| *PHLDA3* \| *STK17B* \| \| *ATP6V1A* \| *EDNRB* \| *JAK2* \| *PHTF2* \| *STK39* \| \| *ATP6V1B2* \| *EDRF1* \| *JUN* \| *PIAS3* \| *STMN1* \| \| *ATP8B2* \| *EEF1A1* \| *JUNB* \| *PIGR* \| *STOM* \| \| *ATP9B* \| *EEF1A2* \| *JUND* \| *PIK3R1* \| *STX18* \| \| *ATXN2L* \| *EEF2* \| *JUP* \| *PIK3R2* \| *STX8* \| \| *AUH* \| *EFHD1* \| *KAT2B* \| *PIK3R3* \| *SULF2* \| \| *AURKA* \| *EFHD2* \| *KAT5* \| *PIM1* \| *SULT1A1* \| \| *AURKB* \| *EFNA1* \| *KCNA1* \| *PIM3* \| *SULT1A3* \| \| *B2M* \| *EFNB2* \| *KCNB1* \| *PITX3* \| *SULT1E1* \| \| *B3GNT3* \| *EFR3A* \| *KCNC1* \| *PKD2L1* \| *SUMO1* \| \| *BAD* \| *EGF* \| *KCNC4* \| *PKIB* \| *SURF4* \| \| *BAG3* \| *EGFR* \| *KCNE5* \| *PKM* \| *SV2B* \| \| *BAG5* \| *EGLN1* \| *KCNF1* \| *PKMYT1* \| *SV2C* \| \| *BAK1* \| *EGR1* \| *KCNG2* \| *PKP2* \| *SYAP1* \| \| *BARHL2* \| *EGR3* \| *KCNIP3* \| *PLA1A* \| *SYK* \| \| *BAX* \| *EIF2AK2* \| *KCNJ11* \| *PLA2G2A* \| *SYN1* \| \| *BAXA* \| *EIF2S2* \| *KCNJ5* \| *PLA2G2D* \| *SYNE3* \| \| *BCAN* \| *EIF4E* \| *KCNK2* \| *PLA2G4A* \| *SYNPO* \| \| *BCAR3* \| *EIF4G1* \| *KCNN4* \| *PLAT* \| *SYTL2* \| \| *BCHE* \| *EIF5* \| *KCTD20* \| *PLAU* \| *TACC3* \| \| *BCL10* \| *EIF5A* \| *KDM5A* \| *PLAUR* \| *TAGLN* \| \| *BCL2* \| *ELAPOR1* \| *KDR* \| *PLCD1* \| *TAP2* \| \| *BCL2L1* \| *ELAVL1* \| *KEAP1* \| *PLCD4* \| *TBC1D31* \| \| *BCL2L12* \| *ELF3* \| *KIAA0753* \| *PLCG1* \| *TBK1* \| \| *BCL3* \| *ELK1* \| *KIF11* \| *PLD1* \| *TBX21* \| \| *BCL6* \| *ELOVL1* \| *KIF14* \| *PLEC* \| *TCF21* \| \| *BDH1* \| *ELOVL2* \| *KIF15* \| *PLEKHF1* \| *TCF4* \| \| *BDKRB2* \| *ELOVL3* \| *KIF20A* \| *PLEKHF2* \| *TCP11* \| \| *BDNF* \| *ELOVL5* \| *KIF23* \| *PLIN4* \| *TCRA* \| \| *BEAN1* \| *EMG1* \| *KIF2C* \| *PLK1* \| *TDO2* \| \| *BEX2* \| *EN2* \| *KISS1* \| *PLK4* \| *TERT* \| \| *BEX4* \| *ENC1* \| *KIT* \| *PLOD2* \| *TFAP2A* \| \| *BGLAP* \| *ENO1* \| *KL* \| *PLOD3* \| *TFAP2C* \| \| *BHLHE40* \| *ENO2* \| *KLF4* \| *PLPP2* \| *TFDP1* \| \| *BID* \| *ENO3* \| *KLF5* \| *PLPPR2* \| *TFF1* \| \| *BIRC2* \| *ENPP1* \| *KLHDC7A* \| *PLXDC1* \| *TFF3* \| \| *BIRC5* \| *ENPP2* \| *KLHL18* \| *PLXNA2* \| *TFPI* \| \| *BMERB1* \| *ENTPD3* \| *KLHL24* \| *PMAIP1* \| *TFRC* \| \| *BMF* \| *EP300* \| *KLHL5* \| *PMEPA1* \| *TG* \| \| *BMP2* \| *EPB41L1* \| *KLK1* \| *PNN* \| *TGFA* \| \| *BMP2K* \| *EPB41L2* \| *KLK2* \| *PNPO* \| *TGFB1* \| \| *BMP4* \| *EPCAM* \| *KLK3* \| *PNPT1* \| *TGFB2* \| \| *BMP5* \| *EPHA1* \| *KLRK1* \| *PNRC1* \| *TGFB3* \| \| *BMP7* \| *EPHA4* \| *KNSTRN* \| *POLI* \| *TGFBI* \| \| *BMP8B* \| *EPHX1* \| *KPNA2* \| *POMC* \| *TGFBR2* \| \| *BNIP1* \| *EPHX2* \| *KRAS* \| *POMP* \| *TGFBR3* \| \| *BPIFB1* \| *EPOR* \| *KREMEN2* \| *POT1* \| *TGM5* \| \| *BRCA1* \| *EPS15* \| *KRT14* \| *POU5F1* \| *TH* \| \| *BRCA2* \| *ERBB2* \| *KRT15* \| *PPAN* \| *THBS1* \| \| *BRINP1* \| *ERBB3* \| *KRT19* \| *PPARA* \| *THOC2* \| \| *BRINP3* \| *ERCC3* \| *KRT33A* \| *PPARG* \| *THPO* \| \| *BSG* \| *ERCC6* \| *KRT5* \| *PPARGC1A* \| *THRA* \| \| *BTF3* \| *ERCC8* \| *KRTAP10-7* \| *PPARGC1B* \| *THRB* \| \| *BTG1* \| *ERLIN2* \| *KRTAP2-4* \| *PPBP* \| *THRSP* \| \| *BTG2* \| *ERO1B* \| *KYNU* \| *PPIF* \| *THSD4* \| \| *BUB1* \| *ESPL1* \| *L1CAM* \| *PPL* \| *THUMPD1* \| \| *BUB1B* \| *ESPN* \| *LAMB1* \| *PPP1R10* \| *THY1* \| \| *BZW1* \| *ESPNP* \| *LAMB3* \| *PPP1R12A* \| *TICAM1* \| \| *C1QA* \| *ESR1* \| *LAMC1* \| *PPP1R1B* \| *TIFA* \| \| *C1S* \| *ESR2* \| *LAMP1* \| *PPP1R3C* \| *TIMM9* \| \| *C3* \| *ESRRA* \| *LBP* \| *PPT1* \| *TIMP1* \| \| *CA2* \| *ETNK2* \| *LCN2* \| *PRAF2* \| *TIMP2* \| \| *CABP1* \| *EXOC1* \| *LCORL* \| *PRAP1* \| *TIMP3* \| \| *CACNA1S* \| *EXOSC7* \| *LCP2* \| *PRCC* \| *TINF2* \| \| *CACYBP* \| *EYA1* \| *LCT* \| *PRDM8* \| *TJP1* \| \| *CALCA* \| *EYA2* \| *LDAH* \| *PRDX1* \| *TK1* \| \| *CALCR* \| *EYA4* \| *LDHA* \| *PRDX2* \| *TKT* \| \| *CALD1* \| *EZH2* \| *LDHB* \| *PRIM1* \| *TLR1* \| \| *CAMK2D* \| *F13A1* \| *LDLR* \| *PRIM2* \| *TLR2* \| \| *CAMK2N1* \| *F2* \| *LDOC1* \| *PRIMA1* \| *TLR4* \| \| *CAMP* \| *F2R* \| *LEFTY2* \| *PRKAA1* \| *TMA16* \| \| *CANX* \| *F8* \| *LEP* \| *PRKACA* \| *TMC4* \| \| *CAPG* \| *FABP3* \| *LEPR* \| *PRKAG3* \| *TMED10* \| \| *CAPN15* \| *FABP4* \| *LGALS1* \| *PRKAR2A* \| *TMED6* \| \| *CAPN3* \| *FADD* \| *LGALS3* \| *PRKCA* \| *TMEM117* \| \| *CAPN9* \| *FAM107A* \| *LGI3* \| *PRKCD* \| *TMEM132D* \| \| *CAR3* \| *FAM131C* \| *LGR5* \| *PRKCQ* \| *TMEM26* \| \| *CARHSP1* \| *FAM168A* \| *LHB* \| *PRL* \| *TMEM263* \| \| *CARS2* \| *FAM184B* \| *LHCGR* \| *PRMT1* \| *TMEM38B* \| \| *CASP1* \| *FAM241B* \| *LHFPL4* \| *PRMT6* \| *TMEM40* \| \| *CASP10* \| *FAM3D* \| *LHX6* \| *PRNP* \| *TMEM64* \| \| *CASP3* \| *FAM43A* \| *LIF* \| *PROCA1* \| *TMEM97* \| \| *CASP7* \| *FAM83E* \| *LIFR* \| *PROK1* \| *TMPO* \| \| *CASP8* \| *FANCC* \| *LIMK1* \| *PROKR1* \| *TMPRSS2* \| \| *CASP8AP2* \| *FANCI* \| *LIN7A* \| *PROS1* \| *TMSB15A* \| \| *CASP9* \| *FAS* \| *LIPE* \| *PRPF6* \| *TMT1A* \| \| *CAT* \| *FASLG* \| *LITAF* \| *PRPF8* \| *TNF* \| \| *CATSPER4* \| *FASN* \| *LMNA* \| *PRRG4* \| *TNFAIP6* \| \| *CAV1* \| *FASTKD1* \| *LMNB1* \| *PRSS1* \| *TNFRSF10A* \| \| *CAV3* \| *FBN1* \| *LMNB2* \| *PRSS16* \| *TNFRSF10B* \| \| *CBFA2T3* \| *FBXO32* \| *LMOD1* \| *PRSS23* \| *TNFRSF11B* \| \| *CBFB* \| *FBXO5* \| *LMX1A* \| *PRSS27* \| *TNFRSF1A* \| \| *CBR1* \| *FCER1A* \| *LPL* \| *PRSS35* \| *TNFRSF21* \| \| *CBS* \| *FCHO2* \| *LPP* \| *PRSS8* \| *TNFRSF25* \| \| *CBX5* \| *FCMR* \| *LRATD1* \| *PRXL2A* \| *TNFRSF9* \| \| *CBX6* \| *FEN1* \| *LRP4* \| *PSAT1* \| *TNFSF10* \| \| *CBX7* \| *FGF1* \| *LRP8* \| *PSEN2* \| *TNFSF11* \| \| *CCDC88B* \| *FGF12* \| *LRRC3* \| *PSMA2* \| *TNFSF14* \| \| *CCK* \| *FGF2* \| *LRRC52* \| *PSMA3* \| *TNKS1BP1* \| \| *CCKAR* \| *FGFR3* \| *LSM14B* \| *PSMB1* \| *TNNC2* \| \| *CCKBR* \| *FGL1* \| *LTBP2* \| *PSMB10* \| *TNNT3* \| \| *CCL11* \| *FGR* \| *LTBR* \| *PSMB8* \| *TNRC18* \| \| *CCL12* \| *FHL2* \| *LXN* \| *PSMD10* \| *TNS1* \| \| *CCL2* \| *FIG4* \| *LY6D* \| *PSPC1* \| *TNS2* \| \| *CCL3* \| *FKBP4* \| *LY6E* \| *PSTPIP2* \| *TOP2A* \| \| *CCL4* \| *FKBP5* \| *LY75* \| *PTEN* \| *TOP2B* \| \| *CCL8* \| *FLT3LG* \| *LYPD5* \| *PTGER1* \| *TP53* \| \| *CCN2* \| *FMO1* \| *LYRM7* \| *PTGES* \| *TP53I11* \| \| *CCN5* \| *FN1* \| *MACF1* \| *PTGIS* \| *TP53I3* \| \| *CCNA1* \| *FOS* \| *MADD* \| *PTGS1* \| *TP53INP1* \| \| *CCNA2* \| *FOSL1* \| *MAEA* \| *PTGS2* \| *TP63* \| \| *CCNB1* \| *FOXA1* \| *MAOA* \| *PTK2* \| *TPD52L1* \| \| *CCNB2* \| *FOXA2* \| *MAP2K1* \| *PTMA* \| *TPI1* \| \| *CCND1* \| *FOXA3* \| *MAP2K2* \| *PTMS* \| *TPM3* \| \| *CCND2* \| *FOXC2* \| *MAP2K3* \| *PTOV1* \| *TPO* \| \| *CCNE1* \| *FOXD1* \| *MAP2K5* \| *PTPN1* \| *TPR* \| \| *CCNG1* \| *FOXO1* \| *MAP2K6* \| *PTPN11* \| *TPSAB1* \| \| *CCNK* \| *FOXO3* \| *MAP3K1* \| *PTPN21* \| *TPSG1* \| \| *CCNO* \| *FSHB* \| *MAP3K14* \| *PTPRA* \| *TPTE* \| \| *CD14* \| *FST* \| *MAP3K7* \| *PTPRG* \| *TRAPPC8* \| \| *CD163* \| *FSTL1* \| *MAP4* \| *PTPRN* \| *TRDN* \| \| *CD24A* \| *FUT3* \| *MAP4K2* \| *PTPRN2* \| *TRH* \| \| *CD36* \| *FYN* \| *MAPK1* \| *PTPRU* \| *TRIM5* \| \| *CD38* \| *G0S2* \| *MAPK11* \| *PUS1* \| *TRIM9* \| \| *CD44* \| *G6PC3* \| *MAPK13* \| *PWP2* \| *TRIP13* \| \| *CD5L* \| *G6PD* \| *MAPK14* \| *PXDN* \| *TRP53* \| \| *CD68* \| *GADD45A* \| *MAPK3* \| *PXN* \| *TRPM4* \| \| *CD7* \| *GADD45G* \| *MAPT* \| *RAB11B* \| *TSC22D1* \| \| *CD74* \| *GALNT14* \| *MASP1* \| *RAB12* \| *TSC22D3* \| \| *CD80* \| *GALNT15* \| *MASTL* \| *RAB14* \| *TSC22D4* \| \| *CD86* \| *GALNT4* \| *MATN3* \| *RAB18* \| *TSHR* \| \| *CDC25B* \| *GALNT5* \| *MATR3* \| *RAB2A* \| *TSPAN1* \| \| *CDC25C* \| *GALR1* \| *MAX* \| *RAB38* \| *TSPAN12* \| \| *CDC45* \| *GALR3* \| *MAZ* \| *RAC1* \| *TSPO* \| \| *CDC6* \| *GAPDH* \| *MBD2* \| *RACGAP1* \| *TSPYL1* \| \| *CDCA5* \| *GAS6* \| *MBOAT7* \| *RAD51* \| *TTC9C* \| \| *CDH1* \| *GATA3* \| *MC1R* \| *RAD51AP1* \| *TTF2* \| \| *CDH2* \| *GBP2* \| *MCAM* \| *RAD51C* \| *TTK* \| \| *CDH22* \| *GCLC* \| *MCCC2* \| *RAF1* \| *TTLL10* \| \| *CDH6* \| *GCLM* \| *MCM10* \| *RALGAPA1* \| *TTR* \| \| *CDK1* \| *GCNT3* \| *MCM2* \| *RANGAP1* \| *TUBA4A* \| \| *CDK2* \| *GDA* \| *MCM4* \| *RAP1GAP* \| *TUBB6* \| \| *CDK2AP2* \| *GDAP1L1* \| *MCM6* \| *RARRES1* \| *TWIST2* \| \| *CDK4* \| *GDF10* \| *MCM7* \| *RASD2* \| *TWNK* \| \| *CDK5* \| *GDF15* \| *MCPT1* \| *RASGRP1* \| *TXLNA* \| \| *CDK5R2* \| *GDNF* \| *MCPT4* \| *RASSF2* \| *TXN* \| \| *CDK6* \| *GDPD5* \| *MDM2* \| *RB1* \| *TXNL4B* \| \| *CDK8* \| *GDPGP1* \| *ME1* \| *RBBP8* \| *TXNRD1* \| \| *CDKN1A* \| *GFRA1* \| *MECP2* \| *RBFOX3* \| *TYMS* \| \| *CDKN1B* \| *GFRA2* \| *MED13* \| *RBL2* \| *TYR* \| \| *CDKN1C* \| *GGH* \| *MED27* \| *RBM13* \| *TYROBP* \| \| *CDKN2B* \| *GGT1* \| *MELK* \| *RBM17* \| *TYRP1* \| \| *CDKN2C* \| *GH1* \| *MEOX1* \| *RBM22* \| *UAP1* \| \| *CDKN3* \| *GHR* \| *MET* \| *RBMS1* \| *UBA5* \| \| *CDT1* \| *GHRH* \| *METTL14* \| *RBP4* \| *UBE2C* \| \| *CEACAM1* \| *GINS1* \| *METTL16* \| *RBSN* \| *UBE2D3* \| \| *CEACAM19* \| *GINS2* \| *MFGE8* \| *RCN1* \| *UBE2T* \| \| *CEBPA* \| *GIPC1* \| *MFN2* \| *RDH13* \| *UBQLN4* \| \| *CEBPB* \| *GJA1* \| *MFSD4A* \| *REEP5* \| *UGT1A1* \| \| *CEBPD* \| *GJA3* \| *MGLL* \| *REG3A* \| *UGT1A10* \| \| *CELA2A* \| *GJB2* \| *MGMT* \| *REL* \| *UGT1A3* \| \| *CELF3* \| *GLB1* \| *MGP* \| *RELA* \| *UGT1A7* \| \| *CELSR2* \| *GLI1* \| *MGST3* \| *RELB* \| *UGT1A8* \| \| *CEMIP* \| *GLRX2* \| *MIA* \| *RELL2* \| *UGT1A9* \| \| *CENPA* \| *GLRX5* \| *MIR136* \| *RELN* \| *UGT2B15* \| \| *CENPE* \| *GLS* \| *MIR141* \| *REN* \| *UHRF1* \| \| *CENPF* \| *GLT6D1* \| *MIR17* \| *RERG* \| *ULK1* \| \| *CENPN* \| *GLUL* \| *MIR200C* \| *RET* \| *UNG* \| \| *CENPU* \| *GMNN* \| *MIR20A* \| *RETN* \| *UPK2* \| \| *CEP78* \| *GNAI3* \| *MIR337* \| *RFC3* \| *USF1* \| \| *CES2A* \| *GNAS* \| *MIR34A* \| *RGS13* \| *USP37* \| \| *CFAP44* \| *GNGT2* \| *MIR434* \| *RGS17* \| *USP47* \| \| *CFLAR* \| *GORAB* \| *MIR663A* \| *RHBDF1* \| *USP7* \| \| *CFTR* \| *GOT1* \| *MKI67* \| *RHOBTB1* \| *UTP20* \| \| *CHAC2* \| *GPAM* \| *MKLN1* \| *RHOBTB2* \| *VASN* \| \| *CHAF1A* \| *GPD1* \| *MKNK2* \| *RHOQ* \| *VASP* \| \| *CHCHD1* \| *GPER1* \| *MLKL* \| *RHOU* \| *VAT1* \| \| *CHEK1* \| *GPIHBP1* \| *MLPH* \| *RIF1* \| *VAV1* \| \| *CHEK2* \| *GPM6B* \| *MMD2* \| *RIMKLA* \| *VAV3* \| \| *CHGB* \| *GPR3* \| *MMP1* \| *RIPK1* \| *VCAM1* \| \| *CHODL* \| *GPR83* \| *MMP10* \| *RIPK3* \| *VCL* \| \| *CHRM1* \| *GPRC5A* \| *MMP14* \| *RIPOR2* \| *VCPKMT* \| \| *CHRM3* \| *GPRIN1* \| *MMP16* \| *RLN3* \| *VDAC1* \| \| *CHRNA3* \| *GPSM1* \| *MMP2* \| *RMI2* \| *VDR* \| \| *CHRNA4* \| *GPT* \| *MMP3* \| *RMND1* \| *VEGFA* \| \| *CHRNA5* \| *GPX1* \| *MMP7* \| *RNASEH1* \| *VEGFC* \| \| *CHUK* \| *GPX2* \| *MMP9* \| *RNASET2* \| *VIM* \| \| *CILP* \| *GPX3* \| *MORN2* \| *RNF169* \| *VPS24* \| \| *CILP2* \| *GPX8* \| *MPC1* \| *RNF183* \| *VPS35L* \| \| *CIT* \| *GRB2* \| *MPHOSPH9* \| *RNF4* \| *VTG2* \| \| *CKS1B* \| *GREB1* \| *MPO* \| *RNF8* \| *VWCE* \| \| *CLCA1* \| *GRIA1* \| *MRPL20* \| *RNFT2* \| *VWF* \| \| *CLCN7* \| *GRIN1* \| *MRPL33* \| *ROCK2* \| *VXN* \| \| *CLDN1* \| *GRIN2D* \| *MRPL49* \| *RPL19* \| *WARS1* \| \| *CLDN11* \| *GRK3* \| *MRPS18A* \| *RPL39* \| *WAS* \| \| *CLDN15* \| *GRM3* \| *MSH2* \| *RPS19* \| *WASL* \| \| *CLDN2* \| *GRM8* \| *MSI1* \| *RPS25* \| *WDR3* \| \| *CLU* \| *GRP* \| *MSI2* \| *RPS3A* \| *WDR86* \| \| *CMTR1* \| *GSN* \| *MSMO1* \| *RPS6* \| *WFDC2* \| \| *CNDP2* \| *GSR* \| *MT1* \| *RPS6KA3* \| *WFDC21* \| \| *CNFN* \| *GSS* \| *MT1X* \| *RPS6KB1* \| *WIPF3* \| \| *CNIH2* \| *GSTA1* \| *MTHFD1* \| *RRAGD* \| *WNK2* \| \| *CNN1* \| *GSTA2* \| *MTHFD2* \| *RRM2* \| *WNT10B* \| \| *COG6* \| *GSTK1* \| *MTM1* \| *RRM2B* \| *WNT5A* \| \| *COL12A1* \| *GSTO1* \| *MTMR7* \| *RRP1* \| *WNT9B* \| \| *COL14A1* \| *GSTP1* \| *MTOR* \| *RSPO1* \| *WT1* \| \| *COL18A1* \| *GTF2B* \| *MUC1* \| *RTL10* \| *WWC1* \| \| *COL1A1* \| *GTSE1* \| *MUC16* \| *RTN4* \| *WWC2* \| \| *COL1A2* \| *GUSB* \| *MUG2* \| *RTN4R* \| *XDH* \| \| *COL2A1* \| *GZMB* \| *MUP1* \| *RUNX2* \| *XIAP* \| \| *COL3A1* \| *H2-M10.1* \| *MYBBP1A* \| *RXRA* \| *XIRP1* \| \| *COL5A1* \| *H2AC6* \| *MYBL2* \| *S100A16* \| *XRCC1* \| \| *COL6A1* \| *H2AX* \| *MYBPH* \| *S100A8* \| *YBX1* \| \| *COL6A2* \| *H2AZ1* \| *MYC* \| *S100A9* \| *YIPF5* \| \| *COL6A3* \| *H2BC15* \| *MYCN* \| *S1PR3* \| *YPEL5* \| \| *COL6A6* \| *H2BC21* \| *MYD88* \| *SAC3D1* \| *YWHAE* \| \| *COL8A1* \| *H2BC5* \| *MYF5* \| *SARS1* \| *YWHAQ* \| \| *COL9A3* \| *HADH* \| *MYH10* \| *SAT1* \| *ZBTB12* \| \| *COMT* \| *HAMP* \| *MYH14* \| *SC5D* \| *ZBTB24* \| \| *COMTD1* \| *HAO2* \| *MYH6* \| *SCARB1* \| *ZBTB7A* \| \| *COPB2* \| *HAPLN2* \| *MYH7* \| *SCARB2* \| *ZEB1* \| \| *COQ2* \| *HAS1* \| *MYL12B* \| *SCD* \| *ZFHX3* \| \| *COQ7* \| *HAUS1* \| *MYLK* \| *SCD2* \| *ZFP354A* \| \| *CORO1C* \| *HC* \| *MYLK2* \| *SCPEP1* \| *ZKSCAN2* \| \| *COTL1* \| *HCFC1R1* \| *MYO18A* \| *SCT* \| *ZMYM3* \| \| *COX1* \| *HDAC1* \| *MYOCD* \| *SCTR* \| *ZNF37A* \| \| *COX4I2* \| *HDAC2* \| *MYOD1* \| *SDC1* \| *ZNF703* \| \| *COX5B* \| *HDAC3* \| *NAIP* \| *SDHA* \| *ZNF704* \| \| *CPE* \| *HDAC9* \| *NAP1L4* \| *SDHB* \| *ZNF706* \| \| *CPEB2* \| *HEATR5A* \| *NARF* \| *SDR39U1* \| *ZNF775* \| \| *ZWINT* \|  \|  \|  \|  \| |  |
| --- | --- | --- | --- | --- | --- | --- | --- | --- | --- | --- | --- | --- | --- | --- | --- | --- | --- | --- | --- | --- | --- | --- | --- | --- | --- | --- | --- | --- | --- | --- | --- | --- | --- | --- | --- | --- | --- | --- | --- | --- | --- | --- | --- | --- | --- | --- | --- | --- | --- | --- | --- | --- | --- | --- | --- | --- | --- | --- | --- | --- | --- | --- | --- | --- | --- | --- | --- | --- | --- | --- | --- | --- | --- | --- | --- | --- | --- | --- | --- | --- | --- | --- | --- | --- | --- | --- | --- | --- | --- | --- | --- | --- | --- | --- | --- | --- | --- | --- | --- | --- | --- | --- | --- | --- | --- | --- | --- | --- | --- | --- | --- | --- | --- | --- | --- | --- | --- | --- | --- | --- | --- | --- | --- | --- | --- | --- | --- | --- | --- | --- | --- | --- | --- | --- | --- | --- | --- | --- | --- | --- | --- | --- | --- | --- | --- | --- | --- | --- | --- | --- | --- | --- | --- | --- | --- | --- | --- | --- | --- | --- | --- | --- | --- | --- | --- | --- | --- | --- | --- | --- | --- | --- | --- | --- | --- | --- | --- | --- | --- | --- | --- | --- | --- | --- | --- | --- | --- | --- | --- | --- | --- | --- | --- | --- | --- | --- | --- | --- | --- | --- | --- | --- | --- | --- | --- | --- | --- | --- | --- | --- | --- | --- | --- | --- | --- | --- | --- | --- | --- | --- | --- | --- | --- | --- | --- | --- | --- | --- | --- | --- | --- | --- | --- | --- | --- | --- | --- | --- | --- | --- | --- | --- | --- | --- | --- | --- | --- | --- | --- | --- | --- | --- | --- | --- | --- | --- | --- | --- | --- | --- | --- | --- | --- | --- | --- | --- | --- | --- | --- | --- | --- | --- | --- | --- | --- | --- | --- | --- | --- | --- | --- | --- | --- | --- | --- | --- | --- | --- | --- | --- | --- | --- | --- | --- | --- | --- | --- | --- | --- | --- | --- | --- | --- | --- | --- | --- | --- | --- | --- | --- | --- | --- | --- | --- | --- | --- | --- | --- | --- | --- | --- | --- | --- | --- | --- | --- | --- | --- | --- | --- | --- | --- | --- | --- | --- | --- | --- | --- | --- | --- | --- | --- | --- | --- | --- | --- | --- | --- | --- | --- | --- | --- | --- | --- | --- | --- | --- | --- | --- | --- | --- | --- | --- | --- | --- | --- | --- | --- | --- | --- | --- | --- | --- | --- | --- | --- | --- | --- | --- | --- | --- | --- | --- | --- | --- | --- | --- | --- | --- | --- | --- | --- | --- | --- | --- | --- | --- | --- | --- | --- | --- | --- | --- | --- | --- | --- | --- | --- | --- | --- | --- | --- | --- | --- | --- | --- | --- | --- | --- | --- | --- | --- | --- | --- | --- | --- | --- | --- | --- | --- | --- | --- | --- | --- | --- | --- | --- | --- | --- | --- | --- | --- | --- | --- | --- | --- | --- | --- | --- | --- | --- | --- | --- | --- | --- | --- | --- | --- | --- | --- | --- | --- | --- | --- | --- | --- | --- | --- | --- | --- | --- | --- | --- | --- | --- | --- | --- | --- | --- | --- | --- | --- | --- | --- | --- | --- | --- | --- | --- | --- | --- | --- | --- | --- | --- | --- | --- | --- | --- | --- | --- | --- | --- | --- | --- | --- | --- | --- | --- | --- | --- | --- | --- | --- | --- | --- | --- | --- | --- | --- | --- | --- | --- | --- | --- | --- | --- | --- | --- | --- | --- | --- | --- | --- | --- | --- | --- | --- | --- | --- | --- | --- | --- | --- | --- | --- | --- | --- | --- | --- | --- | --- | --- | --- | --- | --- | --- | --- | --- | --- | --- | --- | --- | --- | --- | --- | --- | --- | --- | --- | --- | --- | --- | --- | --- | --- | --- | --- | --- | --- | --- | --- | --- | --- | --- | --- | --- | --- | --- | --- | --- | --- | --- | --- | --- | --- | --- | --- | --- | --- | --- | --- | --- | --- | --- | --- | --- | --- | --- | --- | --- | --- | --- | --- | --- | --- | --- | --- | --- | --- | --- | --- | --- | --- | --- | --- | --- | --- | --- | --- | --- | --- | --- | --- | --- | --- | --- | --- | --- | --- | --- | --- | --- | --- | --- | --- | --- | --- | --- | --- | --- | --- | --- | --- | --- | --- | --- | --- | --- | --- | --- | --- | --- | --- | --- | --- | --- | --- | --- | --- | --- | --- | --- | --- | --- | --- | --- | --- | --- | --- | --- | --- | --- | --- | --- | --- | --- | --- | --- | --- | --- | --- | --- | --- | --- | --- | --- | --- | --- | --- | --- | --- | --- | --- | --- | --- | --- | --- | --- | --- | --- | --- | --- | --- | --- | --- | --- | --- | --- | --- | --- | --- | --- | --- | --- | --- | --- | --- | --- | --- | --- | --- | --- | --- | --- | --- | --- | --- | --- | --- | --- | --- | --- | --- | --- | --- | --- | --- | --- | --- | --- | --- | --- | --- | --- | --- | --- | --- | --- | --- | --- | --- | --- | --- | --- | --- | --- | --- | --- | --- | --- | --- | --- | --- | --- | --- | --- | --- | --- | --- | --- | --- | --- | --- | --- | --- | --- | --- | --- | --- | --- | --- | --- | --- | --- | --- | --- | --- | --- | --- | --- | --- | --- | --- | --- | --- | --- | --- | --- | --- | --- | --- | --- | --- | --- | --- | --- | --- | --- | --- | --- | --- | --- | --- | --- | --- | --- | --- | --- | --- | --- | --- | --- | --- | --- | --- | --- | --- | --- | --- | --- | --- | --- | --- | --- | --- | --- | --- | --- | --- | --- | --- | --- | --- | --- | --- | --- | --- | --- | --- | --- | --- | --- | --- | --- | --- | --- | --- | --- | --- | --- | --- | --- | --- | --- | --- | --- | --- | --- | --- | --- | --- | --- | --- | --- | --- | --- | --- | --- | --- | --- | --- | --- | --- | --- | --- | --- | --- | --- | --- | --- | --- | --- | --- | --- | --- | --- | --- | --- | --- | --- | --- | --- | --- | --- | --- | --- | --- | --- | --- | --- | --- | --- | --- | --- | --- | --- | --- | --- | --- | --- | --- | --- | --- | --- | --- | --- | --- | --- | --- | --- | --- | --- | --- | --- | --- | --- | --- | --- | --- | --- | --- | --- | --- | --- | --- | --- | --- | --- | --- | --- | --- | --- | --- | --- | --- | --- | --- | --- | --- | --- | --- | --- | --- | --- | --- | --- | --- | --- | --- | --- | --- | --- | --- | --- | --- | --- | --- | --- | --- | --- | --- | --- | --- | --- | --- | --- | --- | --- | --- | --- | --- | --- | --- | --- | --- | --- | --- | --- | --- | --- | --- | --- | --- | --- | --- | --- | --- | --- | --- | --- | --- | --- | --- | --- | --- | --- | --- | --- | --- | --- | --- | --- | --- | --- | --- | --- | --- | --- | --- | --- | --- | --- | --- | --- | --- | --- | --- | --- | --- | --- | --- | --- | --- | --- | --- | --- | --- | --- | --- | --- | --- | --- | --- | --- | --- | --- | --- | --- | --- | --- | --- | --- | --- | --- | --- | --- | --- | --- | --- | --- | --- | --- | --- | --- | --- | --- | --- | --- | --- | --- | --- | --- | --- | --- | --- | --- | --- | --- | --- | --- | --- | --- | --- | --- | --- | --- | --- | --- | --- | --- | --- | --- | --- | --- | --- | --- | --- | --- | --- | --- | --- | --- | --- | --- | --- | --- | --- | --- | --- | --- | --- | --- | --- | --- | --- | --- | --- | --- | --- | --- | --- | --- | --- | --- | --- | --- | --- | --- | --- | --- | --- | --- | --- | --- | --- | --- | --- | --- | --- | --- | --- | --- | --- | --- | --- | --- | --- | --- | --- | --- | --- | --- | --- | --- | --- | --- | --- | --- | --- | --- | --- | --- | --- | --- | --- | --- | --- | --- | --- | --- | --- | --- | --- | --- | --- | --- | --- | --- | --- | --- | --- | --- | --- | --- | --- | --- | --- | --- | --- | --- | --- | --- | --- | --- | --- | --- | --- | --- | --- | --- | --- | --- | --- | --- | --- | --- | --- | --- | --- | --- | --- | --- | --- | --- | --- | --- | --- | --- | --- | --- | --- | --- | --- | --- | --- | --- | --- | --- | --- | --- | --- | --- | --- | --- | --- | --- | --- | --- | --- | --- | --- | --- | --- | --- | --- | --- | --- | --- | --- | --- | --- | --- | --- | --- | --- | --- | --- | --- | --- | --- | --- | --- | --- | --- | --- | --- | --- | --- | --- | --- | --- | --- | --- | --- | --- | --- | --- | --- | --- | --- | --- | --- | --- | --- | --- | --- | --- | --- | --- | --- | --- | --- | --- | --- | --- | --- | --- | --- | --- | --- | --- | --- | --- | --- | --- | --- | --- | --- | --- | --- | --- | --- | --- | --- | --- | --- | --- | --- | --- | --- | --- | --- | --- | --- | --- | --- | --- | --- | --- | --- | --- | --- | --- | --- | --- | --- | --- | --- | --- | --- | --- | --- | --- | --- | --- | --- | --- | --- | --- | --- | --- | --- | --- | --- | --- | --- | --- | --- | --- | --- | --- | --- | --- | --- | --- | --- | --- | --- | --- | --- | --- | --- | --- | --- | --- | --- | --- | --- | --- | --- | --- | --- | --- | --- | --- | --- | --- | --- | --- | --- | --- | --- | --- | --- | --- | --- | --- | --- | --- | --- | --- | --- | --- | --- | --- | --- | --- | --- | --- | --- | --- | --- | --- | --- | --- | --- | --- | --- | --- | --- | --- | --- | --- | --- | --- | --- | --- | --- | --- | --- | --- | --- | --- | --- | --- | --- | --- | --- | --- | --- | --- | --- | --- | --- | --- | --- | --- | --- | --- | --- | --- | --- | --- | --- | --- | --- | --- | --- | --- | --- | --- | --- | --- | --- | --- | --- | --- | --- | --- | --- | --- | --- | --- | --- | --- | --- | --- | --- | --- | --- | --- | --- | --- | --- | --- | --- | --- | --- | --- | --- | --- | --- | --- | --- | --- | --- | --- | --- | --- | --- | --- | --- | --- | --- | --- | --- | --- | --- | --- | --- | --- | --- | --- | --- | --- | --- | --- | --- | --- | --- | --- | --- | --- | --- | --- | --- | --- | --- | --- | --- | --- | --- | --- | --- | --- | --- | --- | --- | --- | --- | --- | --- | --- | --- | --- | --- | --- | --- | --- | --- | --- | --- | --- | --- | --- | --- | --- | --- | --- | --- | --- | --- | --- | --- | --- | --- | --- | --- | --- | --- | --- | --- | --- | --- | --- | --- | --- | --- | --- | --- | --- | --- | --- | --- | --- | --- | --- | --- | --- | --- | --- | --- | --- | --- | --- | --- | --- | --- | --- | --- | --- | --- | --- | --- | --- | --- | --- | --- | --- | --- | --- | --- | --- | --- | --- | --- | --- | --- | --- | --- | --- | --- | --- | --- | --- | --- | --- | --- | --- | --- | --- | --- | --- | --- | --- | --- | --- | --- | --- | --- | --- | --- | --- | --- | --- | --- | --- | --- | --- | --- | --- | --- | --- | --- | --- | --- | --- | --- | --- | --- | --- | --- | --- | --- | --- | --- | --- | --- | --- | --- | --- | --- | --- | --- | --- | --- | --- | --- | --- | --- | --- | --- | --- | --- | --- | --- | --- | --- | --- | --- | --- | --- | --- | --- | --- | --- | --- | --- | --- | --- | --- | --- | --- | --- | --- | --- | --- | --- | --- | --- | --- | --- | --- | --- | --- | --- | --- | --- | --- | --- | --- | --- | --- | --- | --- | --- | --- | --- | --- | --- | --- | --- | --- | --- | --- | --- | --- | --- | --- | --- | --- | --- | --- | --- | --- | --- | --- | --- | --- | --- | --- | --- | --- | --- | --- | --- | --- | --- | --- | --- | --- | --- | --- | --- | --- | --- | --- | --- | --- | --- | --- | --- | --- | --- | --- | --- | --- | --- | --- | --- | --- | --- | --- | --- | --- | --- | --- | --- | --- | --- | --- | --- | --- | --- | --- | --- | --- | --- | --- | --- | --- | --- | --- | --- | --- | --- | --- | --- | --- | --- | --- | --- | --- | --- | --- | --- | --- | --- | --- | --- | --- | --- | --- | --- | --- | --- | --- | --- | --- | --- | --- | --- | --- | --- | --- |

**Table S5.** GO (Gene Ontology) and KEGG (Kyoto Encyclopedia of Genes and Genomes) pathway enrichment analysis results of genes found to be differentially expressed (*p*-value <0.05, |log2FC| ≥ 1 and |log2FC| ≤ 1) between AD brain tissues compared with adjacent healty tissues. The top 10 significant GO and pathway terms were listed. **MF**; Molecular Function, **BP**; Biological Process, and **CC**; Cellular Component.

|  | Term ID | Term description | *p-*value | *q*-value FDR B&H | Hit in Query List |
| --- | --- | --- | --- | --- | --- |
| MF: Molecular Function | | | | | |
|  | GO:0009055 | electron transfer activity | 2,27E-04 | 1,03E-02 | COX5B, SDHB |
|  | GO:0031072 | heat shock protein binding | 3,93E-04 | 1,03E-02 | ENO1, HSP90AB1 |
|  | GO:0002135 | CTP binding | 4,00E-04 | 1,03E-02 | HSP90AB1 |
|  | GO:0000104 | succinate dehydrogenase activity | 6,01E-04 | 1,03E-02 | SDHB |
|  | GO:0051538 | 3 iron, 4 sulfur cluster binding | 6,01E-04 | 1,03E-02 | SDHB |
|  | GO:0008177 | succinate dehydrogenase (quinone) activity | 6,01E-04 | 1,03E-02 | SDHB |
|  | GO:0017098 | sulfonylurea receptor binding | 6,01E-04 | 1,03E-02 | HSP90AB1 |
|  | GO:0002134 | UTP binding | 8,01E-04 | 1,03E-02 | HSP90AB1 |
|  | GO:0004634 | phosphopyruvate hydratase activity | 8,01E-04 | 1,03E-02 | ENO1 |
|  | GO:0016635 | oxidoreductase activity, acting on the CH-CH group of donors, quinone or related compound as acceptor | 1,00E-03 | 1,03E-02 | SDHB |
| BP: Biological Process | | | | | |
|  | GO:0009060 | aerobic respiration | 1,38E-05 | 3,77E-03 | COX5B,SDHB,ENO1 |
|  | GO:0045333 | cellular respiration | 2,25E-05 | 3,77E-03 | COX5B,SDHB,ENO1 |
|  | GO:0015980 | energy derivation by oxidation of organic compounds | 4,82E-05 | 5,40E-03 | COX5B,SDHB,ENO1 |
|  | GO:0006091 | generation of precursor metabolites and energy | 7,07E-05 | 5,94E-03 | COX5B,SDHB,ENO1 |
|  | GO:0019646 | aerobic electron transport chain | 1,34E-04 | 6,67E-03 | COX5B,SDHB |
|  | GO:0042773 | ATP synthesis coupled electron transport | 1,66E-04 | 6,67E-03 | COX5B,SDHB |
|  | GO:0042775 | mitochondrial ATP synthesis coupled electron transport | 1,66E-04 | 6,67E-03 | COX5B,SDHB |
|  | GO:0006754 | ATP biosynthetic process | 1,85E-04 | 6,67E-03 | SDHB,ENO1 |
|  | GO:0009206 | purine ribonucleoside triphosphate biosynthetic process | 2,29E-04 | 6,67E-03 | SDHB,ENO1 |
|  | GO:0009145 | purine nucleoside triphosphate biosynthetic process | 2,33E-04 | 6,67E-03 | SDHB,ENO1 |
| CC: Cellular Component | | | | | |
|  | GO:0098803 | respiratory chain complex | 2,02E-04 | 1,10E-02 | COX5B,SDHB |
|  | GO:1990565 | HSP90-CDC37 chaperone complex | 3,83E-04 | 1,10E-02 | HSP90AB1 |
|  | GO:0043209 | myelin sheath | 6,36E-04 | 1,10E-02 | COX5B,ENO1 |
|  | GO:0000015 | phosphopyruvate hydratase complex | 7,66E-04 | 1,10E-02 | ENO1 |
|  | GO:0045273 | respiratory chain complex II (succinate dehydrogenase) | 7,66E-04 | 1,10E-02 | SDHB |
|  | GO:0030426 | growth cone | 8,30E-04 | 1,10E-02 | ENO1,HSP90AB1 |
|  | GO:0030427 | site of polarized growth | 8,84E-04 | 1,10E-02 | ENO1,HSP90AB1 |
|  | GO:0031967 | organelle envelope | 1,34E-03 | 1,45E-02 | COX5B,SDHB,ENO1 |
|  | GO:0034751 | aryl hydrocarbon receptor complex | 1,53E-03 | 1,48E-02 | HSP90AB1 |
|  | GO:1990917 | ooplasm | 1,91E-03 | 1,67E-02 | HSP90AB1 |

| KEGG Pathway | | | | | | |
| --- | --- | --- | --- | --- | --- | --- |
|  | M7272 | KEGG_PARKINSONS_DISEASE | 4,97E-04 | 5,08E-03 | COX5B,SDHB |  |
|  | M19540 | KEGG_OXIDATIVE_PHOSPHORYLATION | 5,12E-04 | 5,08E-03 | COX5B,SDHB |  |
|  | M16024 | KEGG_ALZHEIMERS_DISEASE | 8,07E-04 | 5,08E-03 | COX5B,SDHB |  |
|  | M13486 | KEGG_HUNTINGTONS_DISEASE | 9,68E-04 | 5,08E-03 | COX5B,SDHB |  |
|  | M48990 | KEGG_MEDICUS_VARIANT_MUTATION_CAUSED_ABERRANT_HTT_TO_ELECTRON_TRANSFER_IN_COMPLEX_II | 1,40E-03 | 5,89E-03 | SDHB |  |
|  | M47810 | KEGG_MEDICUS_PATHOGEN_ARSENIC_TO_ELECTRON_TRANSFER_IN_COMPLEX_II | 1,68E-03 | 5,89E-03 | SDHB |  |
|  | M47942 | KEGG_MEDICUS_REFERENCE_CITRATE_CYCLE_SECOND_CARBON_OXIDATION_2 | 2,80E-03 | 8,41E-03 | SDHB |  |
|  | M47686 | KEGG_MEDICUS_REFERENCE_ELECTRON_TRANSFER_IN_COMPLEX_IV | 5,32E-03 | 1,24E-02 | COX5B |  |
|  | M47687 | KEGG_MEDICUS_VARIANT_MUTATION_CAUSED_ABERRANT_ABETA_TO_ELECTRON_TRANSFER_IN_COMPLEX_IV | 5,60E-03 | 1,24E-02 | COX5B |  |
|  | M47811 | KEGG_MEDICUS_ENV_FACTOR_ARSENIC_TO_ELECTRON_TRANSFER_IN_COMPLEX_IV | 5,88E-03 | 1,24E-02 | COX5B |  |

**Table S6.** Brain region specific differential expression score values of the elite genes.

| **BRAİN REGİON** | **LOG2 FOLDCHANGE** | **P-VALUE** | **FDR** |
| --- | --- | --- | --- |
| **COX5B: cytochrome c oxidase subunit 5B** | | | |
| Entorhinal Cortex | -0.413 | 1.449e-4 | 5.648e-3 |
| Hippocampus | -0.256 | 5.895e-4 | 0.015 |
| Temporal Cortex | -0.526 | 1.157e-5 | 4.562e-4 |
| Frontal Cortex | -0.14 | 0.009 | 0.046 |
| **ENO1: enolase 1** | | | |
| Entorhinal Cortex | -0.092 | 0.495 | 0.666 |
| Hippocampus | -0.019 | 0.849 | 0.932 |
| Temporal Cortex | -0.455 | 0.025 | 0.097 |
| Frontal Cortex | -0.007 | 0.909 | 0.945 |
| **HSP90AB1: heat shock protein 90kDa alpha family class B member 1** | | | |
| Entorhinal Cortex | -0.597 | 4.587e-3 | 0.037 |
| Hippocampus | -0.287 | 0.032 | 0.155 |
| Temporal Cortex | -0.922 | 1.754e-6 | 1.482e-4 |
| Frontal Cortex | -0.131 | 0.095 | 0.215 |
| **SDHB: succinate dehydrogenase complex iron sulfur subunit B** | | | |
| Entorhinal Cortex | -0.165 | 0.089 | 0.226 |
| Hippocampus | -0.231 | 0.024 | 0.132 |
| Temporal Cortex | -0.515 | 1.686e-3 | 0.013 |
| Frontal Cortex | -0.057 | 0.335 | 0.5 |


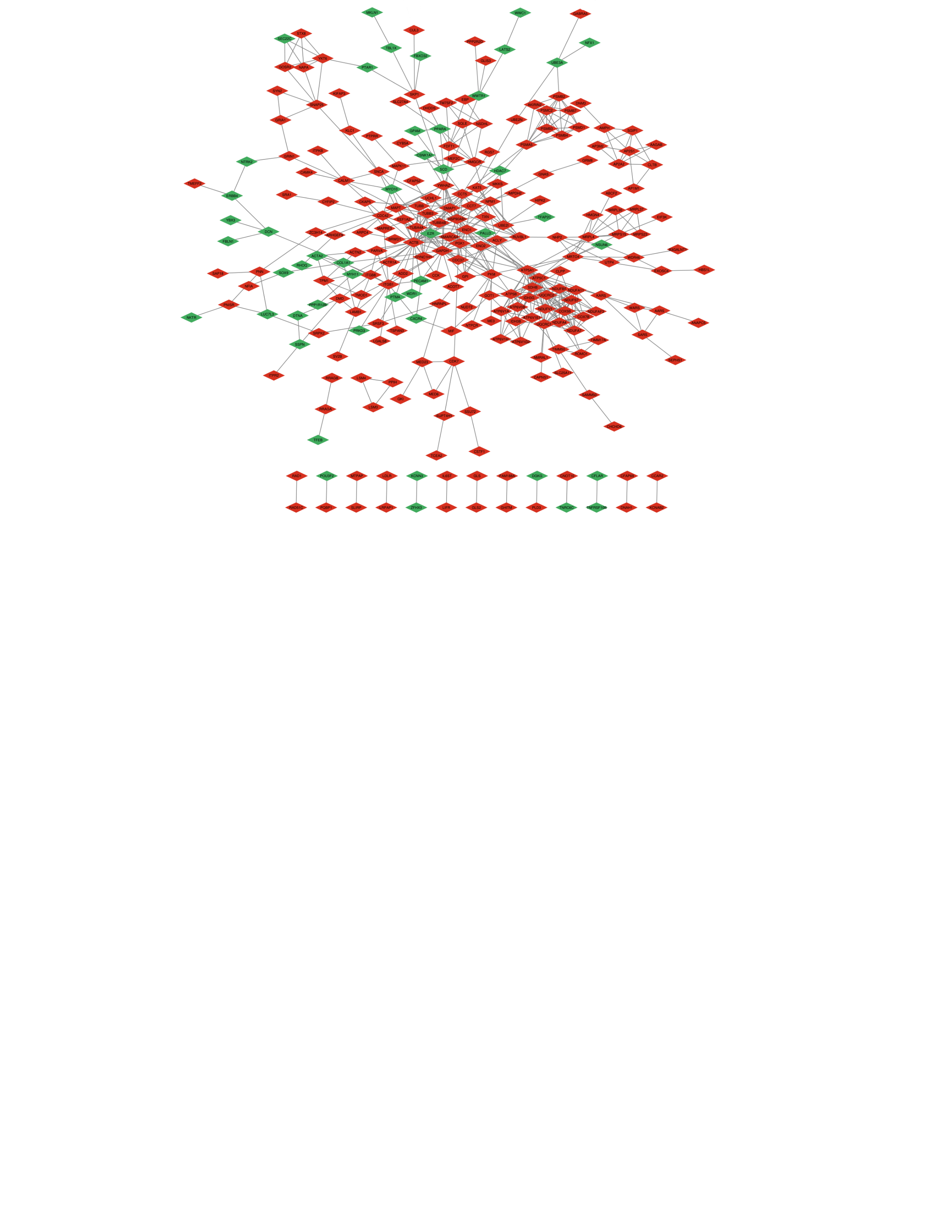


**Figure S1.** The whole protein-protein interaction (PPI) network of DEGs (green nodes represent upregulated DEGs and red nodes represent downregulated DEGs).
